# Serum Peptidomic Analysis in a Comparative Study of the Efficacy of Benjakul Remedy Versus Loratadine in Allergic Rhinitis Patients

**DOI:** 10.3390/ijms27167389

**Published:** 2026-08-18

**Authors:** Vilailak Tiyao, Sittiruk Roytrakul, Janthima Jaresitthikunchai, Sawanya Charoenlappanit, Narumon Phaonakrop, Katanchalee Houngiam, Nichamon Mukkasombut, Pranporn Kuropakornpong, Sunita Makchuchit, Waipoj Chanvimalueng, Neal M. Davies, Arunporn Itharat

**Affiliations:** 1 Graduate School, Faculty of Medicine, Thammasat University, Klongluang, Pathumthani 12120, Thailand; meyamesaya@gmail.com; 2 National Center for Genetic Engineering and Biotechnology, National Science and Technology for Development Agency, Klongluang, Pathumthani 12120, Thailand; sittiruk@biotec.or.th (S.R.); janthima.jar@biotec.or.th (J.J.); sawanya2010@gmail.com (S.C.); narumon.pha@gmail.com (N.P.); 3 Department of Applied Thai Traditional Medicine, Faculty of Medicine, Thammasat University, Klongluang, Pathumthani 12120, Thailand; katanchalee01@gmail.com (K.H.); nichamon.msb@gmail.com (N.M.); 4 Center of Excellence in Applied Thai Traditional Medicine Research (CEATMR), Thammasat University, Klongluang, Pathumthani 12120, Thailand; pranporn.kur@gmail.com (P.K.); msunita_359@hotmail.com (S.M.); 5 Thammasat Postdoctoral Fellowship, Thammasat University, Klongluang, Pathumthani 12120, Thailand; 6 Department of Otolaryngology, Faculty of Medicine, Thammasat University, Klongluang, Pathumthani 12120, Thailand; drwaipoj@gmail.com; 7 Faculty of Pharmacy and Pharmaceutical Sciences, Katz Group-Rexall Centre for Pharmacy and Health Research, University of Alberta, Edmonton, AB T6G 2E1, Canada; ndavies@ualberta.ca

**Keywords:** Benjakul, allergic rhinitis, Thai traditional medicine, serum peptidomics, MALDI-TOF MS, LC-MS/MS, loratadine

## Abstract

Benjakul (BJK) remedy, traditionally used to balance the four elements, has demonstrated clinical efficacy in relieving inflammation and allergic rhinitis (AR) symptoms. This study investigated differential serum peptide expression in AR patients treated with BJK remedy compared to loratadine using MALDI-TOF MS and LC-MS/MS. MALDI-TOF MS revealed four shared mass peptide patterns that were significantly upregulated (*p* < 0.05) after 3 and 6 weeks of both treatments. Identifying the peptides, 17β-hydroxysteroid dehydrogenase (17β-HSD) and microtubule-actin cross-linking factor 1 (MACF1) were linked to inflammatory suppression and hormonal balance, while zinc finger CCCH-type containing 4 (ZC3H4) and transducin beta-like protein 3 (TBL3) were associated with the relief of pulmonary fibrosis. Conversely, zinc finger homeobox 3 (ZFHX3) and transformation/transcription domain-associated protein (TRRAP) remained elevated after treatment, potentially promoting persistent inflammation via E2F transcription factor 1- (E2F1-) and E2F transcription factor 4- (E2F4-) mediated transcription. Among 1113 peptides identified by LC-MS/MS (selected using a significance threshold of *p* < 0.05, without a fixed fold-change cutoff), 6-week treatment with BJK remedy significantly downregulated UBQLN1 (4.562-fold; *p* = 0.016), suppressing toll-like receptor (TLR) activation and B-cell proliferation. It also upregulated Prostaglandin E synthase 2 (PTGES2) and PDZ and LIM domain protein 2 (PDLIM2) (7.841-fold and 8.697-fold, respectively; *p* = 0.004 and 0.002), while enhancing antioxidant and immunoregulatory responses via ATP-binding cassette subfamily B member 8 (ABCB8) and ADP-ribosylation factor GTPase-activating protein 3 (ARFGAP3). By comparison, 3 weeks loratadine treatment significantly downregulated E2F transcription factor 3 (E2F3) and general transcription factor IIIC subunit 6 (GTF3C6) (*p* = 0.034 and 0.017) and upregulated CTD small phosphatase like 2 (CTDSPL2), suggesting a distinct but complementary anti-inflammatory mechanism. Together, these findings enhance the understanding of AR pathophysiology and may help to elucidate the mechanisms of BJK remedy and loratadine in treatment, supporting the further development of targeted therapies and biomarkers.

## 1. Introduction

Allergic rhinitis (AR) is inflammation of the nasal mucosa that occurs when the immune system responds to allergens, most commonly affecting the upper respiratory tract [1]. After exposure to allergens, nasal epithelial cells secrete inflammatory cytokines that activate macrophages and recruit immune cells into the nasal mucosa; these in turn activate plasma cells to produce immunoglobulin E (IgE), which binds receptors on basophils and mast cells, releasing histamine and leukotrienes upon repeated exposure. These mediators cause tissue swelling and lead to nasal stuffiness, conjunctivitis, sneezing, rhinorrhea, and a decline in quality of life, with symptom severity classified according to the Allergic Rhinitis and Its Impact on Asthma (ARIA) guidelines [1]. AR is reported to affect up to 40% of the population in some regions, with its prevalence rising worldwide. In Thailand, the prevalence of AR exceeds 44% [2].

According to Thai traditional medicine (TTM), allergic rhinitis is believed to result from an imbalance in the body’s elements. Specifically, loss of the fire and wind elements leads to the accumulation of the water element and obstruction of the nasal cavity, producing nasal swelling through damage to the earth element. Hot, spicy herbs are traditionally used to promote blood circulation. Benjakul (BJK) remedy, a Thai traditional formula, is recorded in TTM texts as a remedy attributed to the hermit Muratathon and used for more than two thousand years. BJK was originally formulated to restore balance among the five elements (the four classical body elements of Thai traditional medicine, earth, water, wind, and fire, together with air as a fifth, each ascribed to one of the remedy’s five herbs) and has long been used to treat water-element conditions (such as allergies) and earth-element conditions (such as cancer and bone or joint disorders). BJK consists of five hot, spicy herbs: the fruits of *Piper retrofractum* Vahl (syn. *Piper chaba* Hunt.) (earth element), the roots of *Piper sarmentosum* Roxb. (water element), the stems of *Piper interruptum* Opiz. (wind element), the roots of *Plumbago indica* L. (fire element), and the rhizomes of *Zingiber officinale* Roscoe (air element). Piperine, plumbagin, 6-gingerol, 6-shogaol, and myristicin were identified as key bioactive compounds in this remedy and may synergistically contribute antioxidant, antibacterial, anti-inflammatory, antipyretic, and sedative effects [3]. Piperine, plumbagin, 6-gingerol, and 6-shogaol suppress cytokine production through suppressing NFκB and MAPK signaling, whereas myristicin reduces mTOR signaling via the inhibition of its phosphorylation [4,5,6,7]. Moreover, BJK remedy has previously been reported to exhibit anticancer, anti-inflammatory, and anti-allergic activities [8,9]. It reduced inflammation and allergic reactions in an allergy-induced rat model, as shown by ELISA [10]; in a clinical trial, it relieved AR symptoms and improved quality of life without adverse effects [11]; and it has been shown to ameliorate inflammation and pain in osteoarthritis [12]. On this basis, we sought to investigate the mechanisms by which BJK extract may modulate inflammation- and allergy-related protein expression in AR patients. The aim of the present study was therefore to characterize differential serum peptide expression and to identify peptides associated with the efficacy of BJK extract, compared with loratadine (positive control) and a healthy control group, using MALDI-TOF MS and LC-MS/MS. These findings could help to define the proteomic pathway mechanisms by which BJK acts in allergic rhinitis.

## 2. Results

### 2.1. Demographic and Baseline Clinical Characteristics

Forty-six allergic rhinitis patients from a previous study [11], who had completed the full treatment course with either the BJK remedy extract or loratadine, were included, with 23 patients in each arm. The mean age and sex distribution were similar in the two AR groups (Table 1). Healthy participants had a mean age of approximately 30 years, similar to the AR patients. Patients in both the BJK and loratadine groups had mean total nasal symptom score (TNSS) values greater than 5 (6.59 ± 1.86 and 7.17 ± 1.46, respectively; Table 1).

### 2.2. Serum Peptidome Profile Analysis and Differential Expression

**Figure 1:**
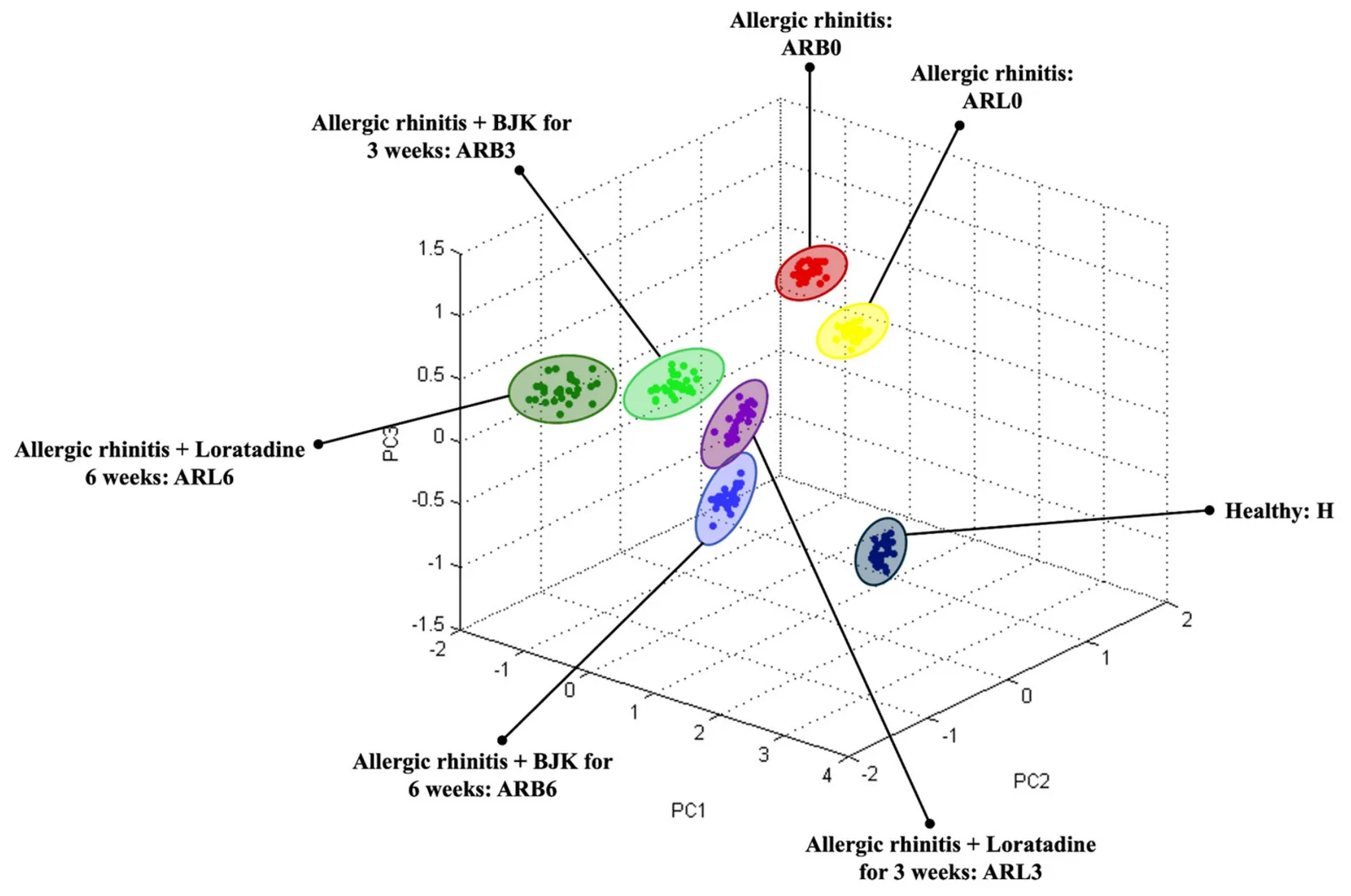
Three-dimensional PCA scatterplot with 95% confidence ellipses added around each treatment/time point cluster (H, ARL0, ARB0, ARL3, ARB3, ARL6, ARB6), illustrating the degree of inter-group separation visible in the peptidome barcode profiles.

**Figure 2:**
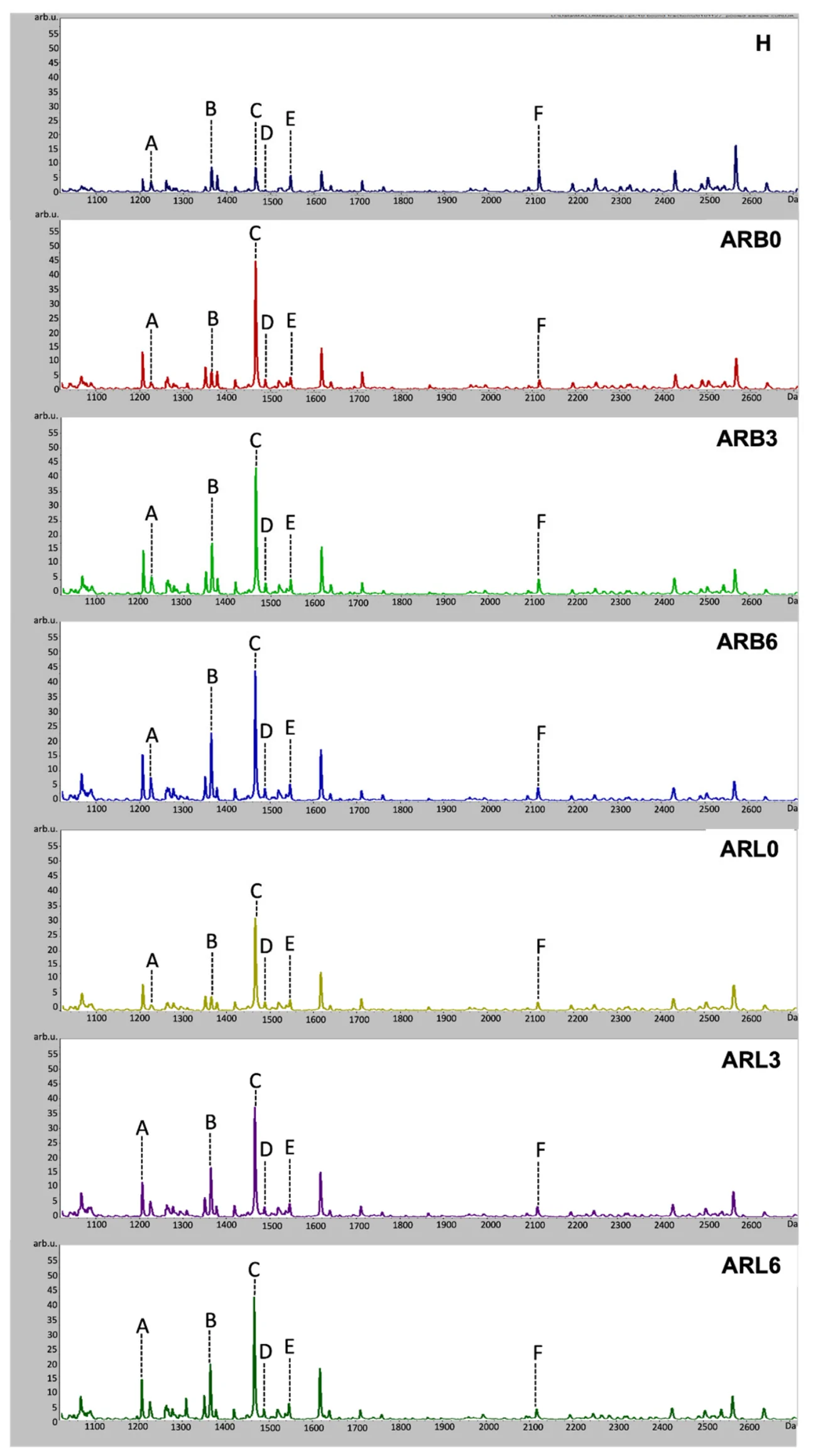
Differential expression of peptide barcodes among healthy group (H), ARL0, ARB0, ARL3, ARL6, ARB3, and ARB6 in range 1000–2500 Da. Candidate peptide mass spectrum. A: 1225.66 Da, B: 1364.31 Da, C: 1465.67 Da, D: 1486.82 Da, E: 1544.21 Da, and F: 2115.84 Da.

Differential mass peptide fingerprints from MALDI-TOF MS were analyzed as distinctive peptide patterns displayed on a three-dimensional principal component analysis (PCA) plot, based on 25 replicate mass spectra per pooled group. The analysis demonstrated uniformity within each group and divergent peptide barcode clusters among healthy participants (H), untreated AR patients (ARL0 and ARB0), patients treated with 10 mg loratadine for 3 and 6 weeks (ARL3 and ARL6), and patients treated with 300 mg BJK for 3 and 6 weeks (ARB3 and ARB6). Each group’s distribution scatterplot showed clear separation; the healthy group (dark blue) was clearly distinguished from the AR groups. The peptide barcode scatterplot of AR patients after 3 and 6 weeks of BJK treatment was separated from that of patients before treatment, consistent with the loratadine arm (Figure 1). The overall cross-validation and recognition capabilities of the peptide barcode scatterplots amounted to 98.06% and 98.86%, respectively, calculated by *t*-test/ANOVA and Wilcoxon/Kruskal–Wallis tests, indicating high confidence. Six candidate peptide masses in the range 1000–2500 Da were detected (Figure 2): 1225.66, 1364.31, 1465.67, 1486.82, 1544.21, and 2115.84 Da. These candidate masses were subsequently subjected to peptide sequencing by LC-MS/MS.

### 2.3. Identification of Candidate Peptide Mass Spectra

**Figure 3:**
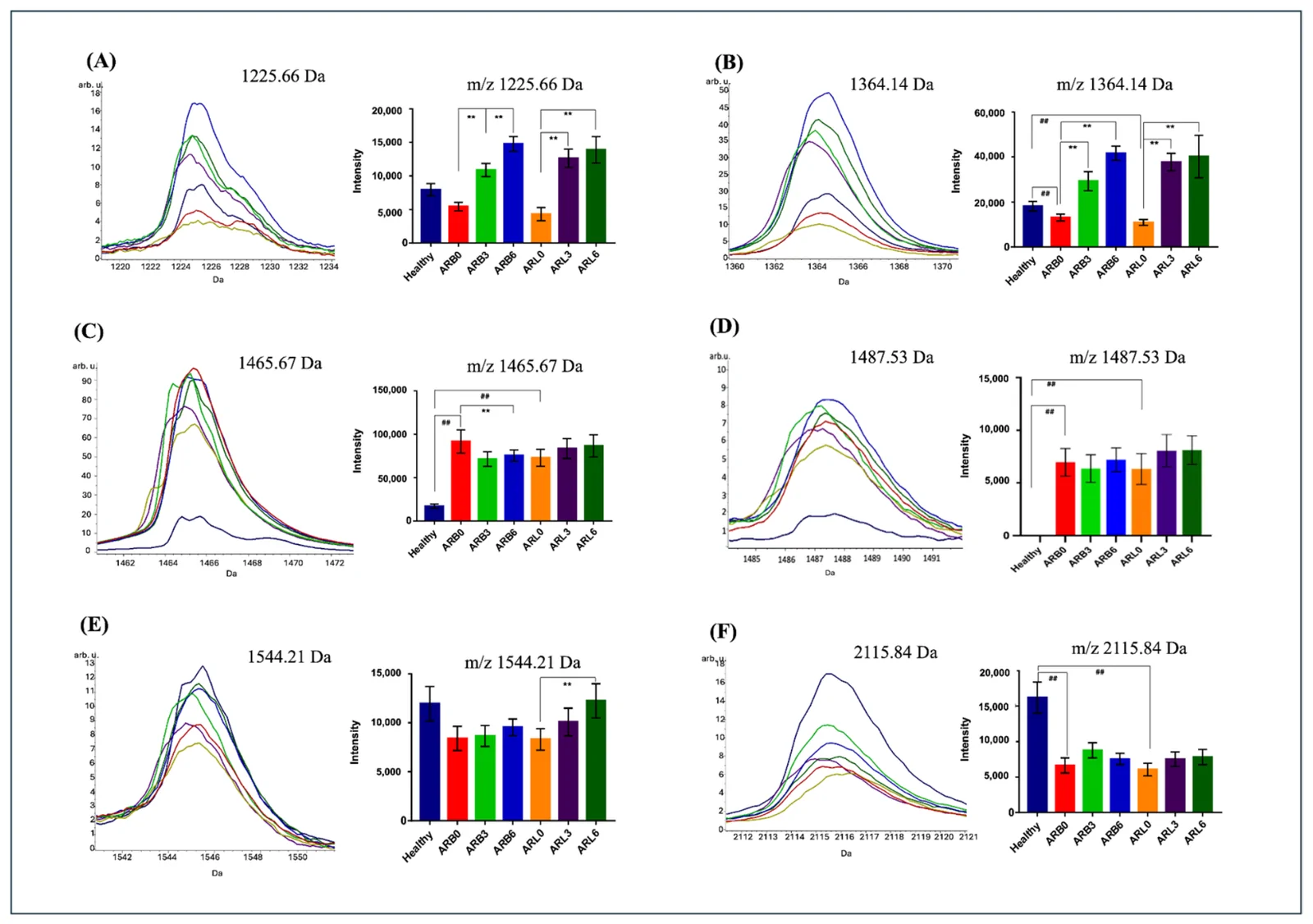
Candidate peptide mass spectra. (**A**) 1225.66 Da, (**B**) 1364.31 Da, (**C**) 1465.67 Da, (**D**) 1486.82 Da, (**E**) 1544.21 Da, and (**F**) 2115.84 Da. A *t*-test/ANOVA and Wilcoxon rank sum test were employed for comparison among these groups, with results considered statistically significant at a *p*-value of less than 0.05. Significant differences between healthy individuals and AR patients are denoted by *p*-values < 0.01 (##) and significant differences between AR patients before and after treatment by *p*-values < 0.01 (**). Note; Color of bar graph which showed the name sample is the same line graph.

Two patterns were observed in the peptide barcodes. In the first pattern, four peptide masses (1225.66, 1364.31, 1544.21, and 2115.84 Da) tended to increase in the healthy group and in AR patients after treatment with both BJK and loratadine. In particular, two pep-tides (1225.66 and 1364.31 Da) showed a significant increase (*p* < 0.01) in the healthy group (H) and in the post-treatment groups (ARL3, ARL6, ARB3, and ARB6) compared with the untreated group (ARL0, ARB0). In the second pattern, peptides at 1465.67 and 1486.82 Da showed significantly increased intensity (*p* < 0.01) in AR patients both before and after treatment compared with healthy individuals (Figure 3). These spectra were further analyzed by LC-MS/MS, and the corresponding peptide sequences were matched against the UniProt database.

The four peptide mass spectra elevated only in the healthy group and in the post-treatment AR groups were identified as 17β-HSD, MACF1, ZC3H4, and TBL3. Their reported functions are associated with the regulation of estrogen production, the Wnt signaling pathway, control of inflammatory cytokine release, and the transcriptional regulation of the TP53 gene (Table 2). The remaining two peptide masses, elevated only in AR patients before and after treatment and not in the healthy group, were identified as ZFHX3 and TRRAP. These peptides have been reported to function in atrial fibrillation and in the transcriptional regulation of inflammatory cytokines (Table 2).

### 2.4. Protein Interaction Networks and Relationships

**Figure 4:**
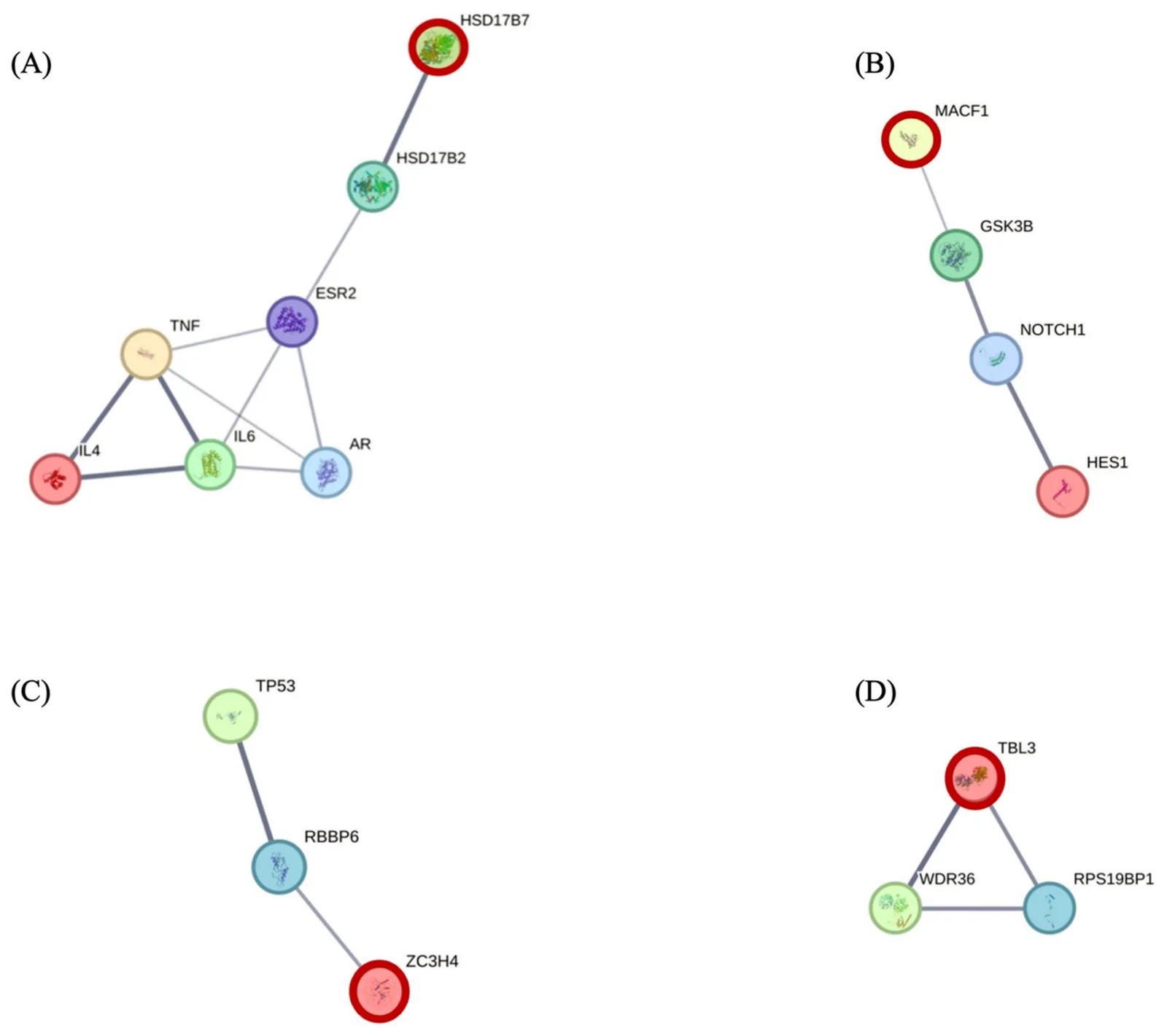
Protein–protein interaction networks of four proteins elevated in healthy individuals and AR patients after treatment: (**A**) HSD17B7, (**B**) MACF1, (**C**) ZC3H4, and (**D**) TBL3 (red circle).

Of the four proteins elevated exclusively in the healthy and post-treatment AR groups (17β-HSD, MACF1, ZC3H4, and TBL3), several have been reported to play roles in controlling inflammation. According to the STRING database, 17β-HSD catalyzes the conversion of estrone to estradiol and DHT to 5α-androstane-3β,17β-diol, playing a key role in maintaining estrogen and androgen homeostasis and in steroid hormone biosynthesis [13]. Through its role in steroid hormone catalysis, 17β-HSD has also been reported to interact with inflammatory cytokine pathways via ESR2 and the androgen receptor (interaction confidence 0.4; grey line, Figure 4A). Both BJK and loratadine increased 17β-HSD levels at 3 and 6 weeks. These results suggest that both treatments may promote the restoration of reproductive-hormone signaling, which is altered in AR patients. Similarly, MACF1 is a cytoskeletal protein that has been implicated in osteogenic bone formation through Wnt and Notch signaling [14]. MACF1 was reduced in AR patients but was elevated after treatment with either drug, suggesting that this protein may be downregulated in AR. MACF1 has been reported to interact with glycogen synthase kinase-3 beta (GSK3B), neurogenic locus notch homolog protein 1 (NOTCH1), and hairy and enhancer of split-1 (HES1) (edge confidence 0.4; black line, Figure 4B). In particular, MACF1 has been shown to delay osteoporosis by suppressing HES1 expression in elderly rats [14]. HES1 also plays a role in mast cell differentiation through the activation of the Notch signaling pathway [19]. Although no current reports link MACF1 directly to AR or inflammation, blocking HES1 may contribute to symptom reduction in AR. ZC3H4 participates in cell death and recycling processes in cancer cells, particularly through interactions with retinoblastoma-binding protein 6 (RBBP6) and TP53 (interaction confidence 0.7; thick black line, Figure 4C). It has also been reported to inhibit pulmonary fibrosis [17]. TBL3 engages in the regulation of ribosome biogenesis and is associated with WD repeat-containing protein 36 (WDR36) and ribosomal protein S19 binding protein 1 (RPS19BP1) [18]; WDR36 has been implicated in periodontal ligament stem cell activity [19]. The TBL3 interaction has the highest edge confidence score (0.9; Figure 4D). Although no prior reports link TBL3 to allergic or inflammatory conditions, it is associated with the regulation of tumor suppressor pathways and T-cell proliferation.

**Figure 5:**
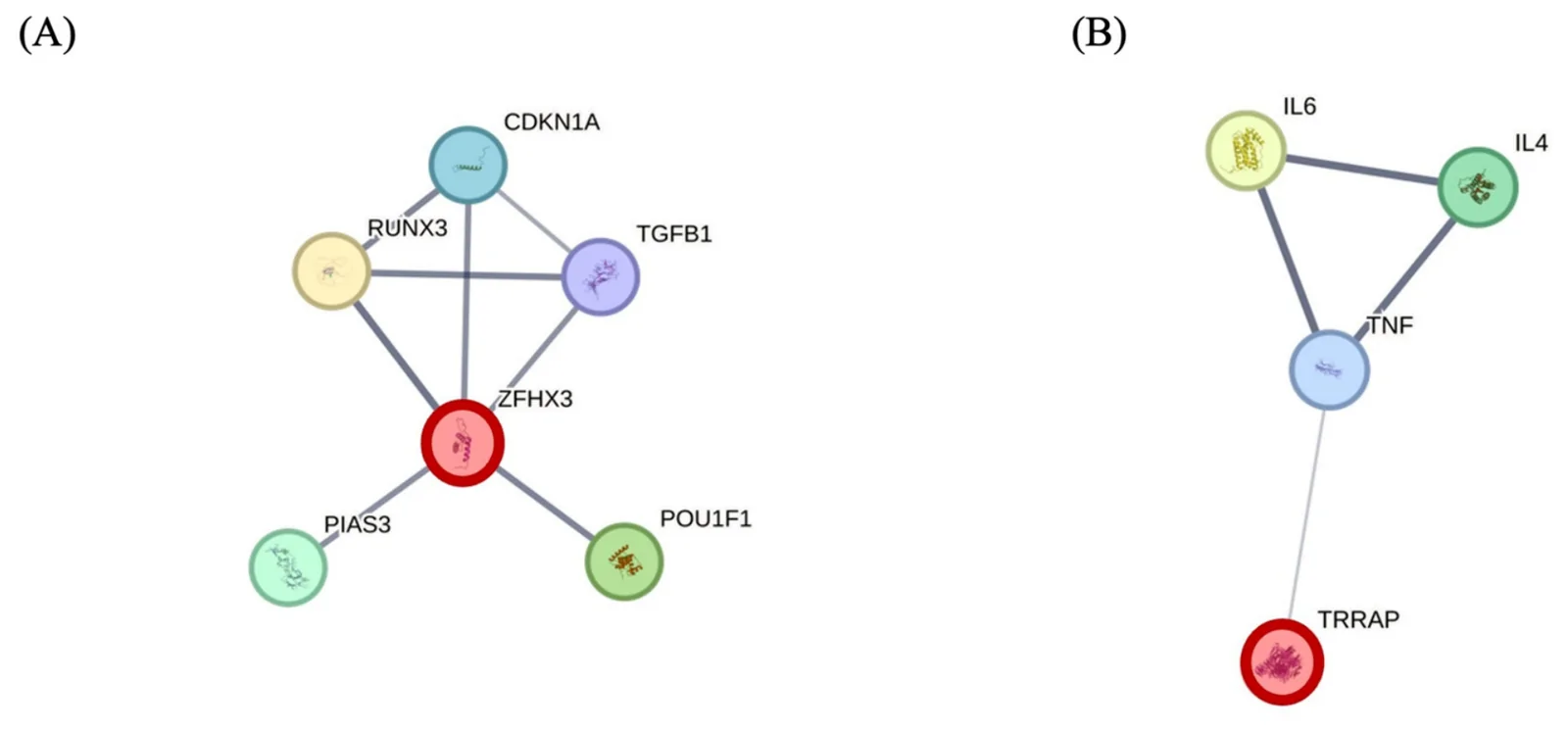
Protein–protein interaction networks of two proteins elevated in AR patients before and after treatment: (**A**) ZFHX3 and (**B**) TRRAP (red circle).

ZFHX3 and TRRAP, by contrast, were elevated in AR patients both before and after treatment, compared with the healthy group. Their interaction networks involve transforming growth factor-beta (TGF-β) signaling and inflammatory cytokine pathways. ZFHX3 is implicated in inflammation through interleukin-11 (IL-11) signaling and the transcriptional regulation of cyclin-dependent kinase inhibitor 1A (CDKN1A) [20,21] and is connected to runt-related transcription factor 3 (RUNX3) and POU class 1 homeobox 1 (POU1F1) (highest edge confidence 0.9; Figure 5A). Owing to its association with CDKN1A, ZFHX3 may contribute to cytokine release during inflammation; it has also been reported as a marker of atrial fibrillation [15]. To our knowledge, no studies have linked ZFHX3 to AR; it may therefore be considered a candidate factor contributing to the persistence of AR symptoms. However, a slight reduction in its expression was observed after treatment with BJK remedy, indicating that this remedy may exert anti-inflammatory effects through the downregulation of this protein. TRRAP interacts with cytokine signaling components including tumor necrosis factor-alpha (TNF-α), IL-4, and IL-6 (medium edge confidence 0.4; Figure 5B). It also co-regulates the transcription of MYC, TP53, E2F1, and E2F4 [16] and has been reported to repress type I interferon-stimulated genes [22]. Although several of its interaction partners have no direct link to inflammation or allergy, they may nevertheless be associated with AR symptom persistence.

### 2.5. Comparison of Peptide Profile Expression Before and After Treatment in AR Using LC-MS/MS

**Figure 6:**
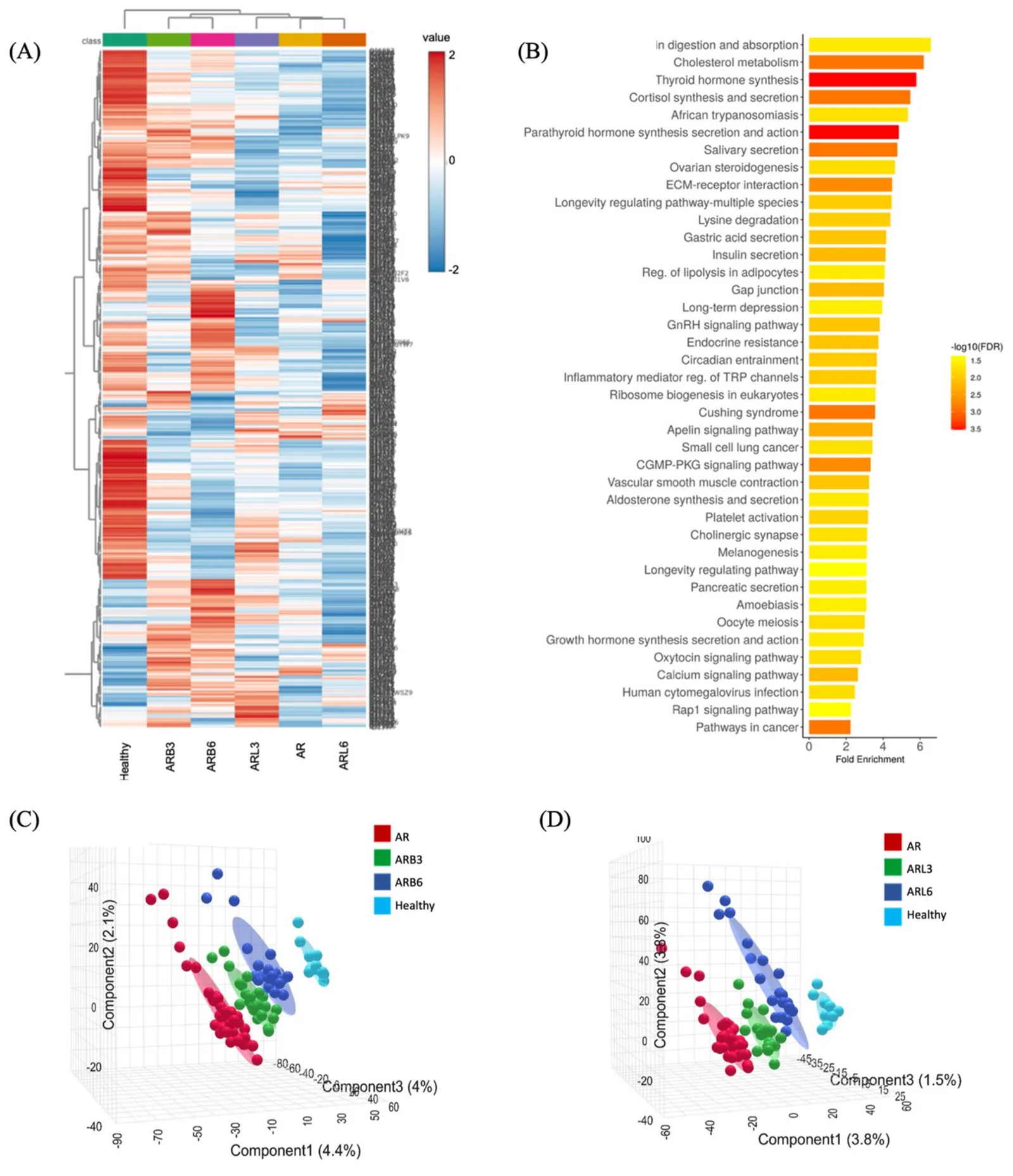
Heatmap of overall peptide expression: upregulated (red) and downregulated (blue) (**A**). Pathway classification of identified proteins based on −log10 (FDR) (**B**). Peptide pattern changes between periods before and after treatment with BJK and loratadine, shown as three-dimensional plots (**C**,**D**).

In addition to the distinctive peptide masses identified above, differential expression analysis revealed a total of 1113 peptides identified by LC-MS/MS and matched to corresponding proteins in the UniProt database. Overall peptide expression showed differential intensity patterns, displayed as a heatmap (upregulated peptides in red, downregulated peptides in blue; Figure 6A). Functional pathway categorization using ShinyGO version 0.8 indicated the significant association of these proteins with several pathways, presented as bar plots based on −log10(FDR) and fold enrichment (Figure 6B). Pathways included vitamin digestion and absorption, cholesterol metabolism, thyroid and parathyroid hormone synthesis, and cortisol secretion (red and orange bars; Figure 6B). Three-dimensional plots were used to visualize the peptide intensity profiles in AR patients treated with BJK remedy and loratadine. The first plot displayed the distinct patterns of healthy participants (H), untreated AR patients (ARB0), and BJK-treated AR patients at 3 weeks (ARB3) and 6 weeks (ARB6); the second plot showed the distributions for the healthy group and AR patients before and after 3 (ARL3) and 6 weeks (ARL6) of loratadine treatment. Both groups showed clear separation between treated and pre-treatment samples, and the peptide patterns at 3 and 6 weeks of treatment moved closer to those of the healthy group in both arms (Figure 6C,D), indicating that protein expression was modulated in a manner consistent with the symptom relief reported in the clinical trial [11].

### 2.6. Classification of Unique Peptide Expression Before and After Treatment

**Figure 7:**
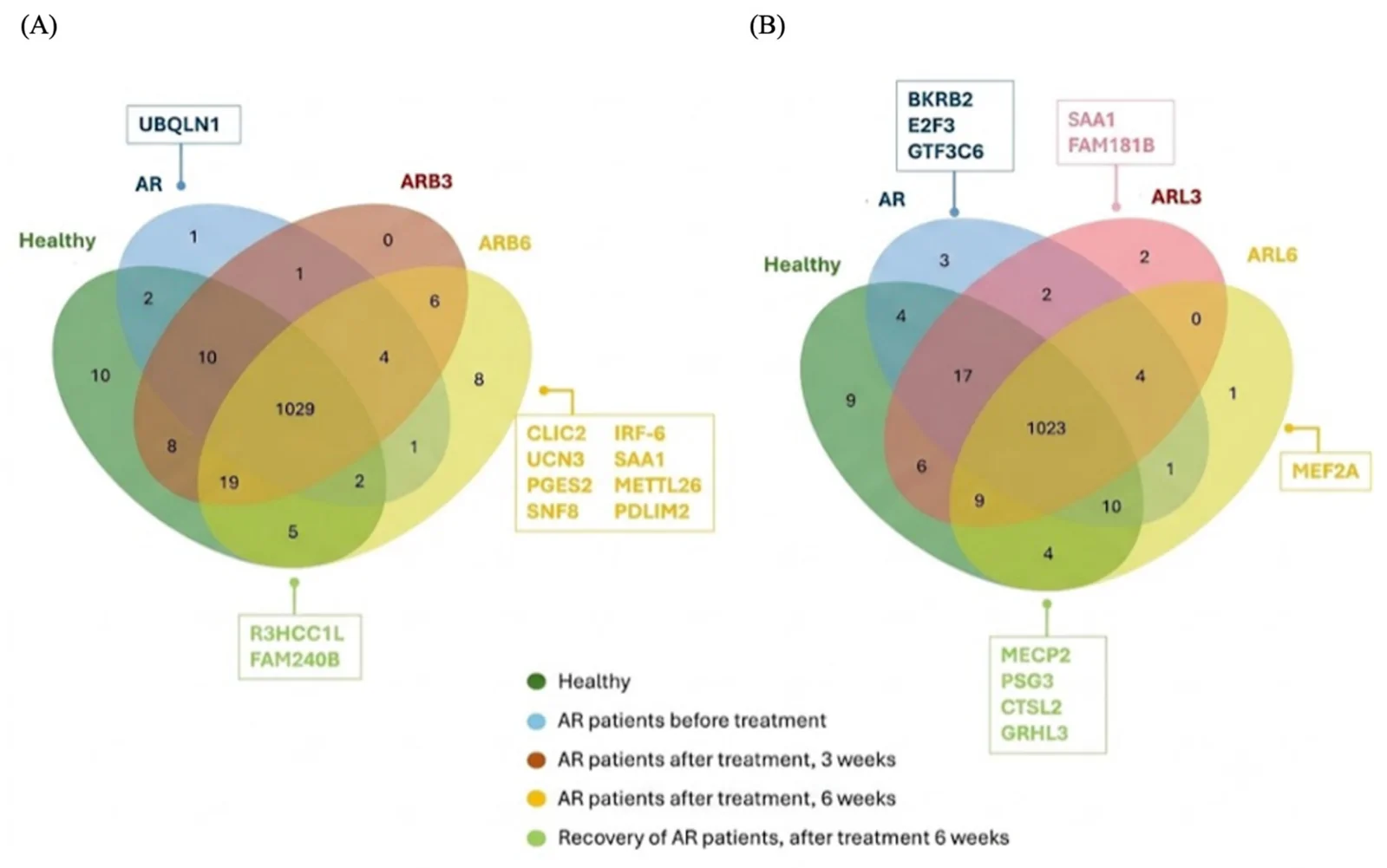
Venn diagrams of peptides detected in each group. (**A**) BJK arm: healthy, AR (pre-treatment), ARB3 (3 weeks post-treatment), and ARB6 (6 weeks post-treatment); 1029 peptides were common to all four groups, with UBQLN1 unique to the pre-treatment AR group and CLIC2, IRF-6, UCN3, SAA1, PGES2, METTL26, SNF8, and PDLIM2 unique to the ARB6 group. (**B**) Loratadine arm: healthy, AR (pre-treatment), ARL3 (3 weeks post-treatment), and ARL6 (6 weeks post-treatment); 1023 peptides were common to all four groups, with BKRB2, E2F3, and GTF3C6 unique to the pre-treatment AR group and MEF2A unique to the ARL6 group.

Because the peptide patterns changed after treatment, Venn diagrams were used to classify the differentially expressed unique peptides in each group, as well as those shared between groups. In the comparison among the healthy group and AR patients before and after BJK treatment, UBQLN1 was the only protein detected exclusively in the pre-treatment group and not after treatment (blue area, Figure 7A). UBQLN1 is localized on the membranes of the endoplasmic reticulum and mitochondria and helps to shuttle mitochondrial proteins to the proteasome for degradation [23,24] (Table 3); it is also involved in B-cell proliferation. Although no unique proteins were observed after 3 weeks of BJK treatment, eight unique peptides were identified after 6 weeks: CLIC2, UCN3, PTGES2, SNF8, IRF6, SAA1, METTL26, and PDLIM2 (yellow area, Figure 7A). Of these, CLIC2 and PDLIM2 are reported to play roles in tumor suppression [25,26], while PTGES2, IRF6, and UCN3 participate in the regulation of inflammation and oxidative stress [27,28,29]. SNF8 and SAA1 are associated with protein transport and autophagy in antiviral defense and have been implicated in balancing inflammation [30,31]. METTL26 is associated with RNA methylation, but its specific biological function remains unclear [32]. Five peptides were shared between the healthy group and the 6-week BJK post-treatment group: ADCK5, ABCB8, ARFGAP3, MPP4, and MAD2L1BP (light green area, Figure 7A). ADCK5, ABCB8, and MAD2L1BP have been described as mitigating oxidative stress, maintaining mitochondrial homeostasis, and regulating cell proliferation [33,34,35]. MPP4 and ARFGAP3 are not directly associated with inflammation reduction; rather, MPP4 engages in maintaining photoreceptor polarity and ARFGAP3 in regulating COPI-mediated vesicular protein transport [36,37]. Details of these peptides are provided in Table 3.

In the loratadine treatment comparison, three peptides were uniquely identified in pre-treatment samples: BKRB2, E2F3, and GTF3C6 (blue area, Figure 7B). These proteins are involved in promoting the release of inflammatory mediators and in cancer cell proliferation [38,39,40]. They were downregulated in the post-treatment groups. Two unique peptides, SAA1 and FAM181B, were elevated after 3 weeks of loratadine treatment (pink area, Figure 7B). SAA1 expression after 3 weeks of loratadine was similar to that observed at 6 weeks of BJK treatment, and it is related to inflammation regulation [31] (see Table 4). At 6 weeks, SAA1, BKRB2, E2F3, and GTF3C6 were downregulated, whereas myocyte-specific enhancer factor 2A (MEF2A) was notably elevated; MEF2A modulates inflammation by suppressing type I IFN production [41]. Four unique peptides overlapped between the 6-week loratadine post-treatment group and the healthy group, suggesting peptides associated with recovery towards a normal state: MECP2, PSG3, CTDSPL2, and GRHL3 (light green area, Figure 7B). These have been reported to regulate inflammation and to suppress cancer (Table 4).

**Table 3:** Classification of differentially expressed unique peptides between pre- and post-treatment with the BJK remedy.

Mass (Da)	Protein Name	Protein ID	Sequence	Score	Function
**Unique peptides in pre-treatment only**					
1159.13	Ubiquilin-1 (UBQLN1)	Q9UMX0	AESGESGGPPGSQ	77.216	Transport of mitochondrial proteins to the proteasome for degradation [23,24]. Activating B-cell proliferation [23].
**Unique peptides in post-treatment at 6 weeks only**					
1796.06	Chloride intracellular channel protein 2 (CLIC2)	O15247	ADCSLLPKLNIIKVAAK	46.041	Maintaining tight junction integrity between endothelial cells [42]. Inhibiting cancer cell migration [25].
1251.62	Urocortin-3 (UCN3)	Q969E3	FHYLRSRDAS	57.841	Attenuates inflammation and ER stress [29].
643.37	Prostaglandin E synthase 2 (PTGES2)	Q9H7Z7	AAALALGG	73.874	Regulates Treg cells and inhibits T-cell receptor signaling [27].
2594.32	Vacuolar-sorting protein SNF8 (SNF8)	Q96H20	AEAKYKERGTVLAEDQLAQMSKQ	45.217	Component of ESCRT-II complex required for autophagy [30].
1726.82	Interferon regulatory factor 6 (IRF-6)	O14896	AIRLCQCKVYWSGPC	106.55	Enhances IFN-β expression and regulates immune response [28].
713.40	Serum amyloid A-1 protein (SAA1)	P0DJI8	AAKRGPGG	41.822	Contributes to and balances inflammation response [31].
806.48	Methyltransferase-like 26 (METTL26)	Q96S19	AAGHLLKP	71.625	RNA methylation [32].
715.34	PDZ and LIM domain protein 2 (PDLIM2)	Q96JY6	ACSPGLPA	85.311	Represses cancer cell progression and decreases inflammatory cytokines production [26].
**Overlapping peptides in healthy group and post-treatment at 6 weeks**					
2313.22	Uncharacterized aarF domain-containing protein kinase 5 (ADCK5)	Q3MIX3	AEKLIKAFAEQIFYTGFIHS	126.49	Associated with coenzyme Q synthesis [33].
829.48	Mitochondrial potassium channel ATP-binding subunit (ABCB8)	Q9NUT2	ALGNVRTV	147.69	Antioxidant activity and reduction of ROS levels [34].
1089.60	ADP-ribosylation factor GTPase-activating protein 3 (ARFGAP3)	Q9NP61	ARLERLSASS	119.17	Component of COPI vesicle coat for protein transportation [37].
2014.00	MAGUK p55 subfamily member 4 (MPP4)	Q96JB8	ACAQLLSAIQKAQEEPQW	179.38	Involved in photoreceptor development [36].
1365.79	MAD2L1-binding protein (MAD2L1BP)	Q15013	ACLRRLFRAIF	78.071	Reduces glioma cell proliferation [35].

**Table 4:** Classification of differentially expressed unique peptides between pre- and post-treatment with loratadine.

Mass (Da)	Protein Name	Protein ID	Sequence	Score	Function
**Unique peptides in only pre-treatment**					
2568.32	B2 bradykinin receptor (BKRB2)	P30411	ACVISYPSLIWEVFTNMLLNVVG	131.99	Stimulates fibroblast proliferation and inflammation in bronchial asthmatics [38].
733.38	Transcription factor E2F3 (E2F3)	O00716	AAAPGAYI	88.55	Promotes inflammatory cytokine release and activates tumor cell growth via activating IL-6, NF-kB, and STAT3 [39].
1704.84	General transcription factor 3C polypeptide 6 (GTF3C6)	Q969F1	CENKCKVLGIDTERP	52.748	Activates cancer cell proliferation and migration [40].
**Unique peptides in only post-treatment at 3 weeks**					
713.40	Serum amyloid A-1 protein (SAA1)	P0DJI8	AAKRGPGG	41.822	See Table 3
793.43	Protein FAM181B (FAM181B)	A6NEQ2	AAPAHGKAA	123.43	Regulates cell development via Hippo signaling pathway [43].
**Unique peptides in only post-treatment at 6 weeks**					
2382.17	Myocyte-specific enhancer factor 2A (MEF2A)	Q02078	APQRPPSTGNAGGMLSTTDLTVPN	101.36	Inhibits IFN-γ release [41].
**Overlapping peptides in healthy group and post-treatment at 6 weeks**					
925.60	Methyl-CpG-binding protein 2 (MECP2)	P51608	AIPKKRGR	152.67	Inhibits inflammation via methylation of p300 of IL-6 gene promotor [44,45].
807.37	Pregnancy-specific beta-1-glycoprotein 3 (PSG3)	Q16557	ACSVRNSA	109.72	Reduces inflammation by suppressing Th 17 proliferation and decreases IL-4, IL-5, and TNF-α levels [46].
2382.99	CTD small phosphatase-like protein 2 (CTDSPL2)	Q05D32	APVTPDSGYSSAHAEATYEEDW	171.91	Dephosphorylates SMAD1/3/5 pathway and suppresses BMP signaling [47].
1609.73	Grainyhead-like protein 3 (GRHL3)	Q8TE85	ATKAMMRVNGDDDSV	130.6	Suppresses proinflammatory mediators IL-1a, STAT1, and Tlr3 [48].

### 2.7. Differential Abundance Analysis of Unique Peptides and Protein–Protein Interactions in BJK Remedy Treatment

The comparison of the unique peptide intensities between pre- and post-treatment with BJK remedy showed that seven of fourteen peptides changed significantly after 6 weeks of treatment: UBQLN1, PTGES2, SAA1, METTL26, PDLIM2, ABCB8, and ARFGAP3 (Table 5).

#### 2.7.1. Peptides Uniquely Detected in Pre-Treatment with BJK Remedy

**Figure 8:**
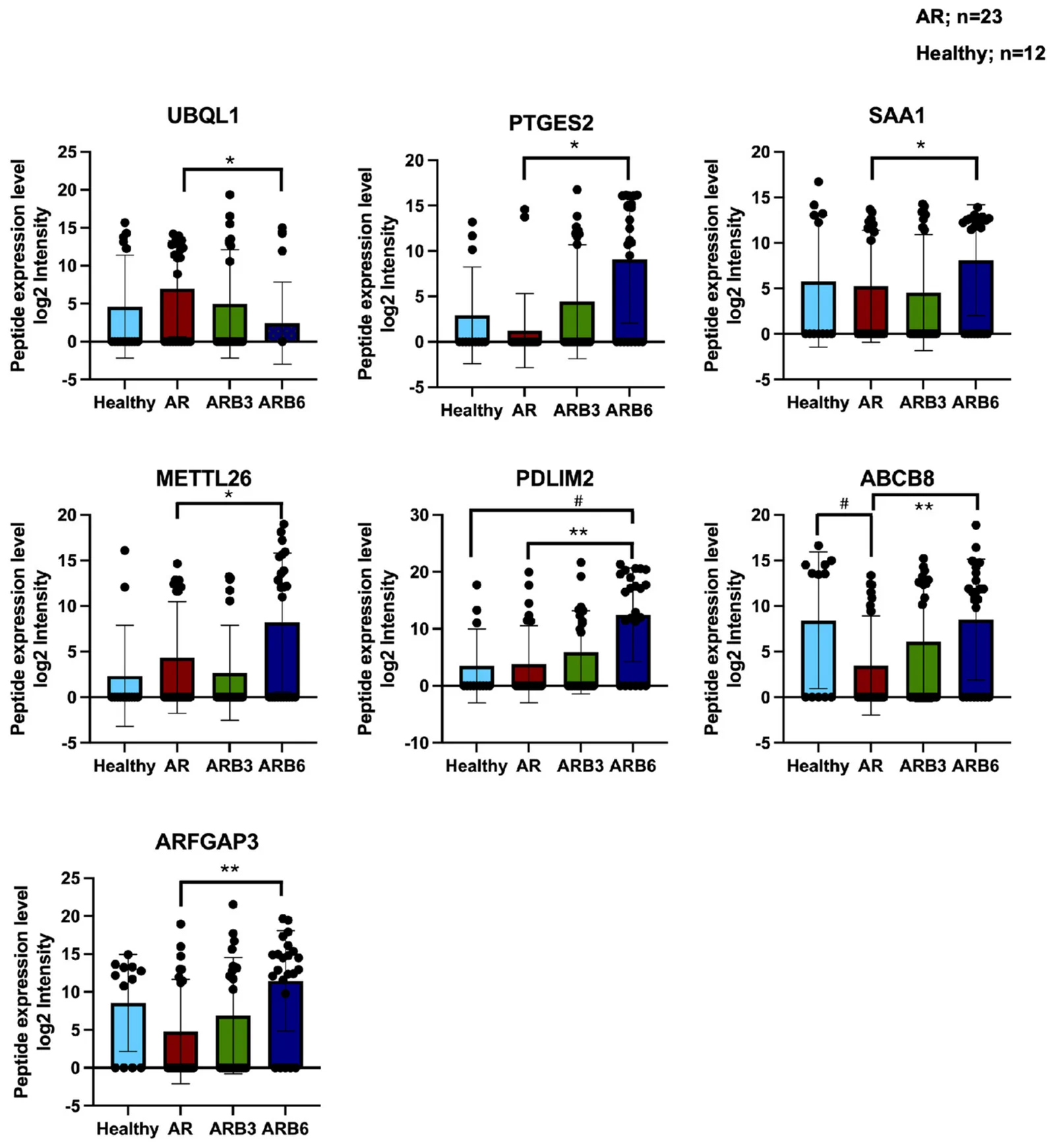
Comparison of significantly differentially expressed unique peptide expression levels among pre-treatment, post-treatment with BJK remedy, and healthy control groups. Data are presented as mean ± SD, with *n* = 23 in AR groups and *n* = 12 in the healthy group. Statistical significance was defined as *p* < 0.05 (*) and *p* < 0.01 (**), based on the Wilcoxon matched-pairs signed-rank test. Additionally, *p* < 0.05 (#) indicated significance according to the Kruskal–Wallis test followed by Dunn’s multiple-comparison test.

**Figure 9:**
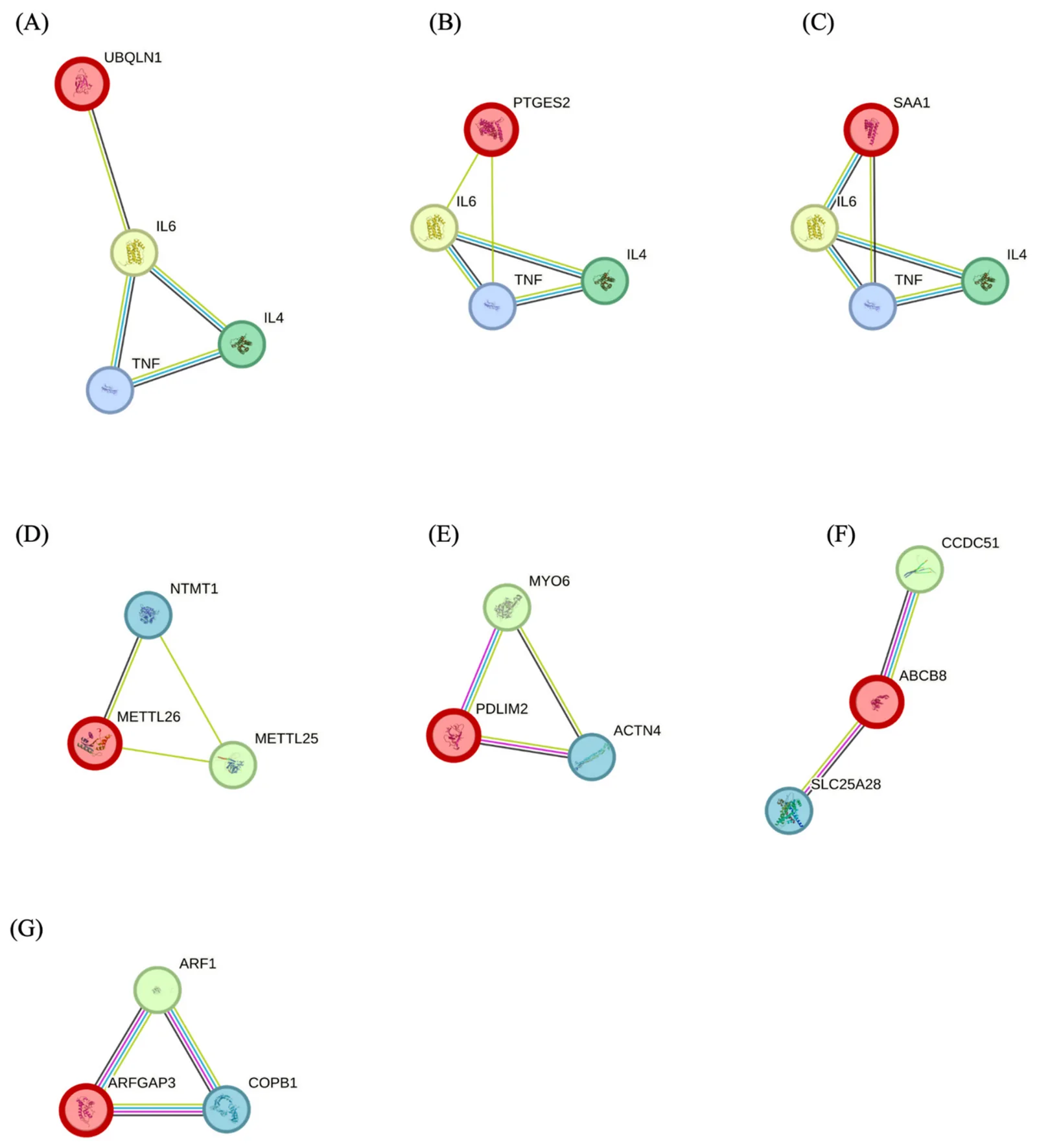
Interaction networks of the 7 significantly differentially expressed unique proteins identified at pre- and post-treatment with BJK remedy (red circle): (**A**) UBQLN1, (**B**) PTGES2, (**C**) SAA1, (**D**) METTL26, (**E**) PDLIM2, (**F**) ABCB8, and (**G**) ARFGAP3. Protein–protein interaction networks were generated using STRING v12.0. Nodes represent individual proteins and are displayed at a uniform size. Colored edges indicate different evidence sources supporting the interaction, including purple = experimentally determined; light blue = from curated database; green = gene neighborhood; red = gene fusion; blue = gene co-occurrence; light green = text mining; black = co-expression; light purple = protein homology. Edge thickness reflects the STRING interaction confidence score, with thicker edges indicating higher-confidence interactions.

UBQLN1 was the only protein that was significantly elevated before treatment compared with the 6-week post-treatment timepoint (Figure 8) and is therefore considered a key candidate potentially involved in driving inflammation and allergic responses in AR. UBQLN1 has been linked to the inflammatory process through mTOR [49] and Toll-like receptor activation on T cells and is predicted to interact with IL-4, IL-6, and TNF (medium edge confidence 0.4; Figure 9A). UBQLN1 has also been reported to support B-cell proliferation via B-cell receptor (BCR) signaling, a key mechanism in the development of allergic responses [23]. The reduced expression of this peptide after treatment, mirroring the level seen in healthy controls, suggests that BJK remedy may relieve allergic symptoms by suppressing UBQLN1 expression.

#### 2.7.2. Peptides Uniquely Detected in Post-Treatment at 6 Weeks with BJK Remedy

Four peptides were significantly increased after 6 weeks of BJK treatment, with expression patterns similar to those of the healthy group despite no significant change after 3 weeks: PTGES2, ABCB8, ARFGAP3, and SAA1. PTGES2, which showed a more than seven-fold increase after treatment, is a key enzyme that converts prostaglandin H2 into prostaglandin E2, a central mediator of inflammation. PTGES2 has been reported to play dual pro- and anti-inflammatory roles depending on the receptor context. In its pro-inflammatory role, PGE2 contributes to classical inflammatory responses such as redness, swelling, and pain, whereas, in its anti-inflammatory role, it can suppress T-cell receptor signaling and IL-2 and IL-4 release from Th2 cells [27] (Figure 9B). PGE2 also inhibits the release of bronchoconstriction mediators from mast cells and decreases eosinophil recruitment in asthma [50]. ABCB8 was upregulated more than five-fold compared with pre-treatment (Table 5; Figure 8) and was also elevated in the healthy group. ABCB8 localizes to the inner mitochondrial membrane and acts as an antioxidant by binding and reducing intracellular ROS levels [34]. It has been reported to interact with CCDC51 and SLC25A28 in maintaining mitochondrial homeostasis (highest edge confidence 0.9; Figure 9F). ARFGAP3 was significantly upregulated—more than three-fold—compared with pre-treatment (Table 5; Figure 8). ARFGAP3 is involved in vesicular transport to the lysosome and interacts with COPB1 and ARF1 (highest confidence 0.9; Figure 9G). Although it has been reported to support glucose uptake by myofibroblasts during muscle injury repair [51], with no direct evidence linking it to inflammation, it may help to mitigate oxidative stress and mitochondrial dysfunction in skeletal muscle [52]. SAA1 was significantly upregulated—more than two-fold—after 6 weeks of treatment compared with pre-treatment (Figure 8; Table 5). The pre-treatment expression of SAA1 was comparable to that of the healthy group. SAA1 is principally released during acute-phase inflammation [31] and is linked to IL-4, IL-6, and TNF (highest edge confidence 0.9; Figure 9C). The increased expression observed at 6 weeks suggests a potential role in immune regulation and homeostatic adjustment.

#### 2.7.3. Shared Peptides Detected at 6 Weeks Post-Treatment and in Healthy Group

PDLIM2 and METTL26 were significantly elevated—more than three- and eight-fold, respectively—in the 6-week BJK group compared with pre-treatment (Figure 8). Their levels were even higher than those of the healthy group. PDLIM2 regulates NF-κB and STAT activity to suppress cancer metastasis and activates NAD(P)H quinone oxidoreductase 1 to suppress oxidative stress and block TLR-mediated innate immune signaling, thereby attenuating IL-6 and IL-12 release [26]. It also interacts with ACTN4 in supporting cell adhesion (highest confidence 0.9; Figure 9E). The elevated expression of PDLIM2 compared with the healthy group suggests that BJK administration may further enhance PDLIM2 levels and contribute to redox balance and ROS clearance. METTL26 participates in RNA methylation and may regulate immune responses, oxidative stress, and inflammasome activity, although its precise function remains incompletely defined [32]; it interacts with METTL25 and NTMT1 (medium edge confidence 0.4; Figure 9D). Its elevated expression at 6 weeks suggests a potential role in post-therapeutic adjustment and tissue remodeling. Several peptides were detected in only a subset of samples, reflecting individual variability in peptide abundance. The detection frequencies for each peptide are summarized in Table 5.

### 2.8. Differential Abundance Analysis of Unique Peptides and Protein–Protein Interactions in Loratadine Treatment

The comparison of the unique peptide intensities between AR patients pre- and post-treatment with loratadine showed that three of ten peptides (E2F3, GTF3C6, and CTDSPL2) changed significantly after 3 and/or 6 weeks of treatment (Table 6).

#### 2.8.1. Peptides Uniquely Detected in Pre-Treatment with Loratadine

**Figure 10:**
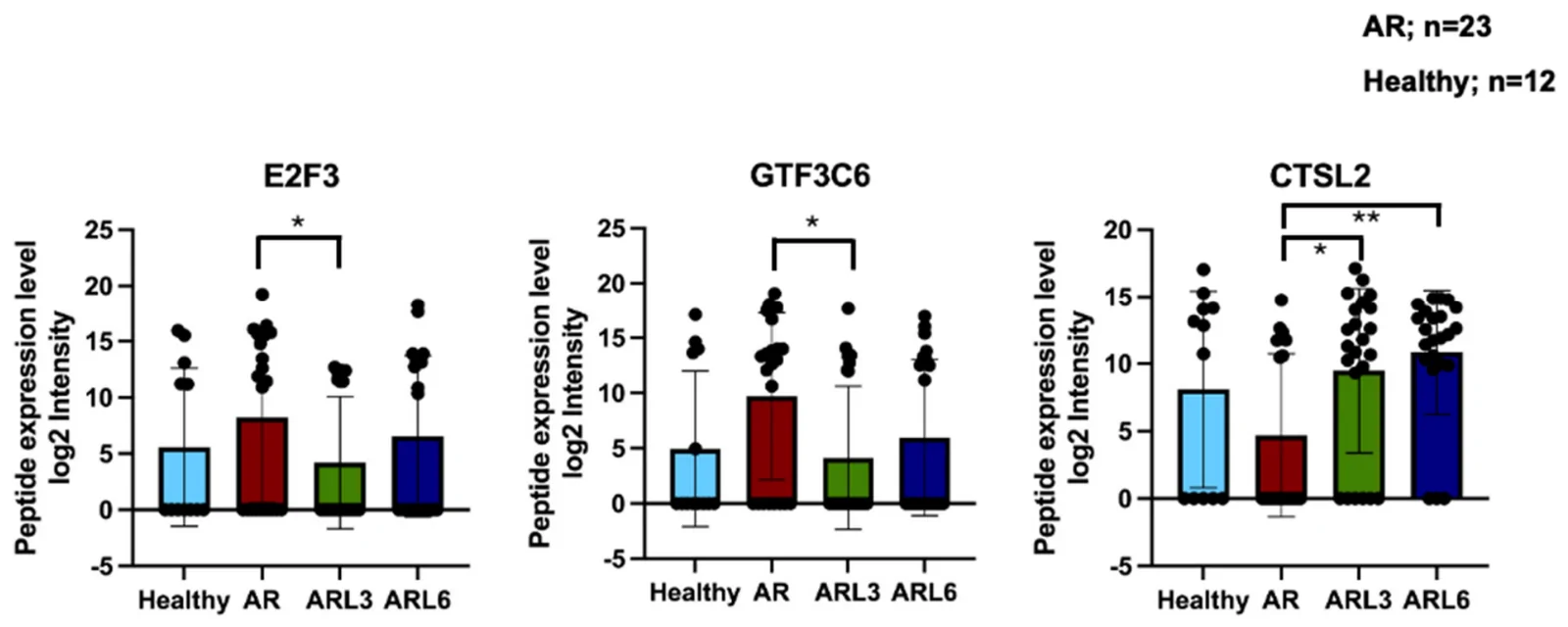
Comparison of significantly differentially expressed unique peptide intensities among pre-treatment, post-treatment with loratadine, and healthy control groups. Data are presented as mean ± SD, with *n* = 23 in AR groups and *n* = 12 in the healthy group. Statistical significance was defined as *p* < 0.05 (*) and *p* < 0.01 (**), based on the Wilcoxon matched-pairs.

**Figure 11:**
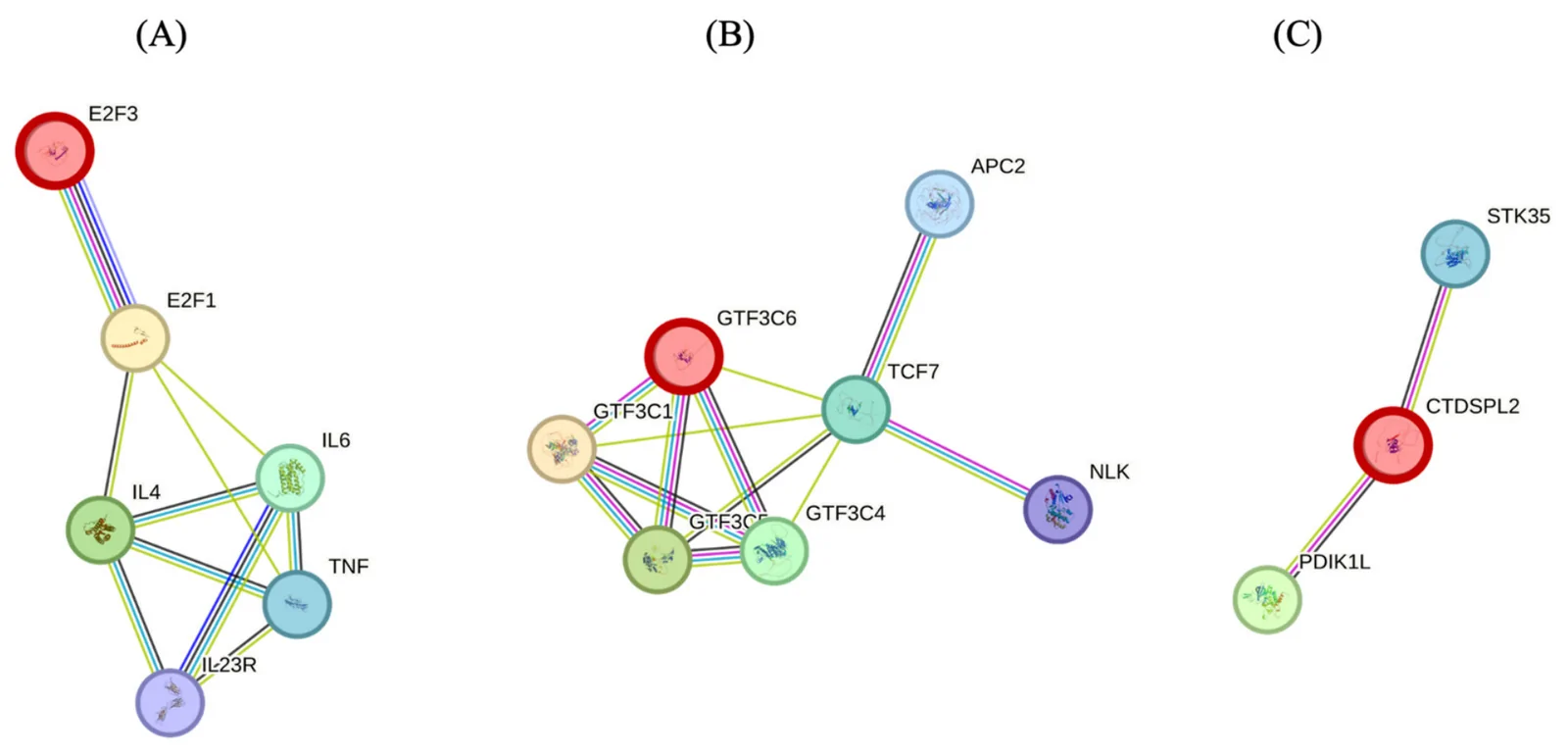
Interaction networks of the three significantly differentially expressed unique proteins identified at pre- and post-treatment with loratadine (red circle): (**A**) E2F3, (**B**) GTF3C6, (**C**) CTDPL2. Protein–protein interaction networks were generated using STRING v12.0. Nodes represent individual proteins and are displayed at a uniform size. Colored edges indicate different evidence sources supporting the interaction, including purple = experimentally determined; light blue = from curated database; green = gene neighborhood; red = gene fusion; blue = gene co-occurrence; light green = text mining; black = co-expression; light purple = protein homology. Edge thickness reflects the STRING interaction confidence score, with thicker edges indicating higher-confidence interactions.

E2F3 and GTF3C6 were more than four- and five-fold higher before treatment than at 3 weeks post-treatment (Figure 10). E2F3 was identified as interacting with E2F1, TNF-α, IL-6, and IL-4 (medium edge confidence 0.4; Figure 11A). E2F3 is critically involved in DNA damage regulation and cancer progression and has been reported to promote inflammation and tumorigenesis via the TNF-α–NF-κB and IL-6–JAK–STAT signaling cascades, notably in nasopharyngeal carcinoma cell models [39]. E2F3 has also been implicated in hepatocellular carcinoma [53]; however, no direct association with allergic rhinitis has been reported to date. GTF3C6 showed an expression trend similar to that of E2F3 and was associated with TCF7 and APC (edge confidence 0.9 and 0.4, respectively; Figure 11B). GTF3C6 plays a role in lung cancer proliferation via the FAK pathway and contributes to IgE-mediated degranulation [40]. After 3 and 6 weeks of loratadine treatment, the peptide levels of both proteins had declined, indicating that loratadine may alleviate AR symptoms by suppressing this mechanism.

#### 2.8.2. Peptides Uniquely Detected at 6 Weeks Post-Treatment with Loratadine

CTDSPL2 levels increased significantly—more than two-fold—after 6 weeks of loratadine treatment (Table 6; Figure 10). CTDSPL2 has been reported to interact with STK35 and PDLIM1 (medium and high edge confidence 04 and 0.7, respectively; Figure 11C) and to dephosphorylate the Smad1/3/5 cascade, reducing inflammatory cytokine release by inhibiting BMP signaling [47]. Although direct links between loratadine and PDLIM1 have not been reported, this protein may serve as a key therapeutic mediator in relieving AR symptoms.

## 3. Discussion

Previous studies have demonstrated the efficacy of BJK remedy in alleviating AR symptoms [11]; however, the protein-level mechanisms underlying its immune-regulatory and tissue repair effects remain largely unclear. We therefore analyzed alterations in specific protein expression before and after treatment with BJK remedy and loratadine to elucidate the biological mechanisms underlying the therapeutic response.

Screening by MALDI-TOF MS revealed distinct alterations in peptide barcode profiles between the pre-treatment and 3- and 6-week post-treatment timepoints. These changes fell into two major patterns: (1) peptides whose intensity increased after treatment to levels comparable with those of healthy controls and were low before treatment and (2) peptides that were predominantly upregulated both before and after treatment but downregulated in the healthy group. The first pattern was represented by 17β-HSD, MACF1, ZC3H4, and TBL3. Notably, 17β-HSD and MACF1 were significantly elevated in the post-treatment groups, approaching the levels seen in healthy individuals. In particular, 17β-HSD contributes to the synthesis of estradiol and 5α-androstane-3β,17β-diol, maintaining estrogen and androgen homeostasis, which in turn modulates inflammation. Previous studies have shown that these hormones suppress the release of inflammatory cytokines such as IL-1β, IL-6, IL-10, TNF, and IFN-γ in activated alveolar macrophages and human endothelial cells [54]. Consistent with previous findings that ginger extract (a BJK constituent) elevates estrogen levels in polycystic ovary syndrome [51,55], the present results suggest that BJK and loratadine may exert therapeutic effects in part by modulating hormone-regulating pathways. MACF1 has been mainly implicated in alleviating osteoporosis through the suppression of HES1 [14], which is associated with the promotion of mast cell proliferation [19]. Consistent with previous reports demonstrating the anti-allergic activity and bone-protective effects of BJK, *Piper sarmentosum* extracts, and plumbagin [4,56], the present findings suggest that this remedy may support bone preservation and immune modulation. Both BJK and loratadine may therefore exert similar therapeutic effects on AR symptoms by upregulating these proteins, and BJK may also regulate AR symptoms through these pathways despite the absence of direct evidence linking it to inhibition of AR. The remaining two peptides, ZC3H4 and TBL3, tended to increase after treatment, although no significant difference was observed compared with pre-treatment. Although no direct evidence supports their roles in attenuating allergic reactions, they have been reported to inhibit pulmonary fibrosis and to control ribosome biogenesis [17,18]. Similarly, plumbagin has been reported to reduce lung fibrosis in mice [57]. These findings suggest that BJK remedy and loratadine may modulate the expression of these peptides to relieve AR symptoms and suppress inflammation, although their direct association with AR remains unreported.

ZFHX3 and TRRAP, by contrast, remained elevated after treatment and are known to promote the release of inflammatory cytokines (e.g., IL-4 and IL-6) by activating the transcription of E2F1 and E2F4 [16]. Nevertheless, ZFHX1 expression was modestly reduced in the post-treatment BJK remedy group, suggesting that the anti-inflammatory effect of BJK remedy may be mediated, at least in part, through the downregulation of this protein. These findings suggest that treatment may alleviate AR symptoms by inhibiting these specific cytokine pathways. However, the sustained expression of ZFHX3 and TRRAP indicates that transcriptional regulators may not be normalized by short-term treatment despite clinical improvement, suggesting the persistence underlying molecular dysregulation. Clinically, this observation raises the possibility that these proteins could serve as biomarkers of persistent disease activity or susceptibility to relapse. Accordingly, the persistent expression of these proteins after treatment is consistent with the chronic nature of AR, in which symptoms can recur upon allergen re-exposure once treatment ceases. Further mechanistic and longitudinal studies are warranted to clarify their roles in disease persistence.

Beyond shared peptide responses, LC-MS/MS revealed distinct expression patterns between BJK and loratadine, highlighting differential mechanisms. UBQLN1 was significantly elevated before treatment and decreased after 3 and 6 weeks of BJK administration. UBQLN1 has been implicated in triggering inflammatory mediator release via TLR activation on T cells and in supporting allergic responses through B-cell proliferation and chemokine secretion via BCR signaling [23,24,49], processes that may be attenuated by treatment. These findings are consistent with previous reports that BJK remedy inhibits TNF-α, IL-1β, and IL-6 expression [8,10]. Notably, 6-shogaol, a bioactive constituent of BJK, exhibits anticancer and anti-inflammatory activities by suppressing cytokine production and inhibiting the PI3K/Akt/NF-κB signaling pathway [19]. The six markers used to standardize the extract are themselves relevant to the activities reported here: piperine, 6-gingerol, and 6-shogaol are well-documented anti-inflammatory constituents, and plumbagin has both anti-inflammatory and bone-protective actions, so, together, these compounds provide a plausible chemical basis for the anti-allergic and anti-inflammatory effects of the remedy [8,58].

In the loratadine arm, E2F3 and GTF3C6 were significantly reduced after 3 weeks, although the effect was not sustained at 6 weeks. These proteins promote inflammation and IgE release through binding with NF-κB and JAK–STAT genes and through the activation of FAK signaling [39,40]. Loratadine has previously been shown to reduce inflammatory cytokine release by attenuating NF-κB signaling and to decrease serum IgE [59]. Both BJK remedy and loratadine therefore exert anti-inflammatory and anti-allergic effects, but through distinct molecular pathways.

Beyond peptide downregulation, our findings revealed protein expression patterns associated with both the resolution of inflammation and tissue remodeling. Three peptides were significantly upregulated in the BJK group, with patterns similar to those in the healthy group: PTGES2, ABCB8, and ARFGAP3. PTGES2, which catalyzes the conversion of PGH2 to PGE2, regulates immune response balance by inhibiting T-cell receptor signaling and thereby suppresses the production of inflammatory mediators [40]. Beyond its anti-inflammatory effect, PTGES2 has been proposed to attenuate bronchoconstriction in asthma through the suppression of mast cell and eosinophil degranulation [48]. Likewise, *Piper sarmentosum* extract has been shown to ameliorate gastric ulcers in rats by increasing PGE2 [60]. BJK remedy also displayed antioxidant activity and attenuated mitochondrial reactive oxygen species (ROS) through the elevated expression of ABCB8, consistent with previous reports that BJK and its constituents reduce oxidative stress and intracellular ROS [10]. The upregulation of ARFGAP3, a vesicular transport protein, was also observed; ARFGAP3 has been reported to contribute to muscle injury repair [51,52], in line with reports that BJK remedy supports bone loss repair [61]. SAA1 was also significantly increased at 6 weeks. As a key regulator of inflammatory responses, SAA1 modulates pro-inflammatory cytokines including IL-4, IL-6, and TNF-α [31]. Although traditionally considered a marker of acute inflammation, SAA1 has more recently been implicated in maintaining immune balance and resolving chronic inflammation. PDLIM2 and METTL26 were also elevated after 6 weeks, with expression levels exceeding those in the healthy group. PDLIM2 may help to reduce the release of inflammatory mediators and inhibit tumor metastasis by suppressing NF-κB, STAT, and TLR signaling [26], and it can attenuate ROS production by stimulating NAD(P)H quinone oxidoreductase 1 [26]. Similarly, piperine has been reported to reduce pain sensation and inflammation by blocking the TRPV1 receptor [62]. Since piper plants constitute more than 60% of the composition of BJK remedy, piperine was found as a significant constituent in the extract and may play an important role in contributing to symptom relief. BJK remedy may also support tissue repair by upregulating METTL26, which participates in cell proliferation and remodeling, although its precise function remains incompletely characterized [32]. These findings are consistent with the clinical efficacy of BJK in osteoarthritis of the knee [12]. In comparison, loratadine promoted CTDSPL2 expression, reducing inflammatory cytokine release through the suppression of BMP signaling. Consistent with previous reports, desloratadine has been shown to suppress heterotopic ossification by dephosphorylating BMP2 and the Smad1/3/5 cascade [63], although no evidence currently links this mechanism to allergic rhinitis. Overall, both BJK and loratadine alleviate AR symptoms through the modulation of specific protein expression, with BJK showing greater involvement in tissue repair and remodeling.

BJK remedy and loratadine appear to share a common mechanism for relieving AR symptoms via the upregulation of 17β-HSD and MACF1, and they may reduce pulmonary fibrosis and support tissue repair through increased ZC3H4 and TBL3. BJK treatment additionally decreased ZFHX3, suggesting an anti-inflammatory effect through the modulation of its expression, whereas TRRAP remained elevated in both groups at levels comparable to the baseline, indicating that symptom recurrence remains likely upon allergen re-exposure, consistent with the absence of a permanent cure for AR. LC-MS/MS analysis further revealed two complementary pathways: BJK reduced inflammation by downregulating UBQLN1 and upregulating PTGES2 and PDLIM2, while promoting antioxidant activity and immune modulation via ABCB8 and ARFGAP3; loratadine, by contrast, suppressed E2F3 and GTF3C6 and increased CTDSPL2, reflecting a distinct but complementary anti-inflammatory mechanism [11].

To our knowledge, this is the first report characterizing the peptidomic mechanisms of BJK and loratadine in AR: both compounds suppressed inflammatory cytokine pathways, but BJK additionally promoted tissue repair and antioxidant activity, with a clinical safety profile comparable to that of loratadine. These findings support the use of BJK for conditions attributed to water- and earth-element imbalances in Thai traditional medicine and justify the validation of these proteins in larger cohorts for targeted therapy or biomarker development. These results also support the use of BJK in the treatment of conditions ascribed to water- and earth-element diseases in Thai traditional medicine and are consistent with previous clinical evidence showing that BJK extract improves allergic rhinitis symptoms with safety comparable to loratadine. Loratadine is also well established as a second-generation antihistamine for allergic rhinitis symptom control [64,65].

A limitation of the present study is that patients were not stratified by sensitization profile (seasonal versus perennial allergic rhinitis) or by baseline disease intensity, and it remains possible that the variable inflammatory burden at enrolment influenced the magnitude of the peptide changes observed; this should be examined prospectively in future work. Background antihistamine treatment was withdrawn for at least one week before sampling as part of the inclusion criteria (Section 4.3), but no other concurrent AR treatments were systematically recorded. Because BJK remedy showed efficacy comparable to loratadine regarding several of the mechanisms assessed here, whether BJK could serve as an add-on to antihistamine therapy is an open and clinically relevant question that this dataset was not designed to address, and this warrants dedicated combination-therapy trials.

## 4. Materials and Methods

### 4.1. Chemicals and Reagents

Ammonium persulfate (Bio Basic Inc. (Markham, ON, Canada)), bovine serum albumin (Sigma-Aldrich, Steinheim, Germany), copper (II) sulfate (Thermo Fisher Scientific, Bremen, Germany), Folin–Ciocalteu reagent (Merck Millipore, Darmstadt, Germany), sodium carbonate (PanReac AppliChem, Darmstadt, Germany), sodium hydroxide (Merck Millipore, Darmstadt, Germany), and tartaric acid (Carlo Erba, Italy) were used for protein concentration measurement using the Lowry assay. Peptide fractionation was performed using acetonitrile (RCI Labscan, Bangkok, Thailand), trifluoroacetic acid (Sigma-Aldrich, Steinheim, Germany), and ZipTip C18 (Merck Millipore, Darmstadt, Germany). For MALDI-TOF MS analysis, α-cyano-4hydrocinnamic acid (CHCA) matrix, acetonitrile, and trifluoroacetic acid (Sigma-Aldrich, Steinheim, Germany) were used, together with the ProteoMass Peptide & Protein MALDI-MS calibration Kit (Sigma-Aldrich, St. Louis, MO, USA). LC-MS/MS analysis was performed using LC-grade acetonitrile (RCI Labscan, Thailand) and formic acid (Fluka, Seelze, Germany).

### 4.2. Preparation of Drug Formulation

The BJK extract used in this study was the standardized ethanolic extract of the five-herb remedy, and its marker content was quantified by reversed-phase high-performance liquid chromatography (HPLC) using the method previously developed and validated by Itharat and Sakpakdeejaroen (2010) [66]. In analytical terms, a validated method is one that has been formally demonstrated to be specific, accurate, precise, and reproducible for the analytes of interest; because the present work used the same standardized extract and the same validated method, the marker content could be determined reliably without repeating the full validation. Six marker compounds were selected for standardization because they are the principal bioactive constituents of the five herbs and are considered responsible for the reported anti-allergic, anti-inflammatory, and cytotoxic activities of the remedy: piperine and methyl piperate, the characteristic amides of the long-pepper species *Piper chaba* (syn. *Piper retrofractum*) and *Piper interruptum*; myristicin from *Piper sarmentosum*; plumbagin from *Plumbago indica*; and 6-gingerol and 6-shogaol from *Zingiber officinale*. Quantified against authentic reference standards, the markers were present at 97.46 ± 6.06 mg/g (piperine), 12.00 ± 0.46 mg/g (6-shogaol), 11.27 ± 0.76 mg/g (6-gingerol), 6.83 ± 0.25 mg/g (myristicin), 5.55 ± 0.40 mg/g (plumbagin), and 3.07 ± 0.15 mg/g (methyl piperate) of BJK extract. Separation was performed on a reversed-phase C18 column with a water–acetonitrile gradient (40–100% acetonitrile over 60 min) at a flow rate of 1.0 mL/min and ultraviolet detection at 256 nm, with each marker being identified by the comparison of its retention time with that of the corresponding reference standard. A representative HPLC chromatogram, in which each major peak is annotated with its marker compound and the herb from which the marker is derived, is provided in Supplementary Figure S1. The BJK and loratadine capsules were prepared as described by Houngiam et al. (2019) [11].

### 4.3. Inclusion Criteria

The serum samples used in the present study were collected during a previous clinical trial [7]; only samples from participants with a clinical history of mild or moderate allergic rhinitis, who had completed the full course of treatment, were included. In addition, these patients had a total nasal symptom score (TNSS) greater than 5 before treatment, in accordance with the Allergic Rhinitis and its Impact on Asthma (ARIA) guidelines, and had no clinical history of disqualifying comorbidities. Participants who had discontinued antihistamine use for at least one week before the study were also included.

### 4.4. Experimental Design and Sample Recruitment

All serum samples were obtained from patients who had completed a full course of allergic rhinitis treatment in a previous clinical trial approved by the Medical Ethics Committee of the Faculty of Medicine, Thammasat University (certificate of approval No. MTU-EC-TM-4-183/57) and registered at ClinicalTrials.gov (identifier NCT03376594). Only the 46 allergic rhinitis patients aged 20–70 years who had completed treatment (samples obtained before treatment and after 3 and 6 weeks of continuous treatment) were included. The TNSS, nasal cavity cross-sectional area, basophil levels, and quality-of-life scores were considered as additional clinical factors. Serum samples were divided into two groups: BJK extract remedy (300 mg/day) and loratadine (10 mg/day) as a positive control. The sample size was calculated using the formula

n = [N·σ^2^·z^2^α/2]/[d^2^(N − 1) + σ^2^·z^2^α/2],

where z(α/2) and z(1 − β) are the standard normal deviates for the significance level and power, respectively (z(α/2) = 1.96 at the 95% confidence level and z(1 − β) = 0.84 for 80% power); d is the maximum allowed error (0.2); σ is the standard deviation derived from the prior study (1.42); N is the total sample size in the previous study (N = 60); and α = 0.05. Accordingly, the required sample size per group was n = 23. Serum samples from healthy participants (n = 12) were also collected for comparison.

### 4.5. Sample Preparation

Protein concentrations in serum samples were measured by the Lowry assay and adjusted to a final concentration of 1 µg/µL. All serum samples were fractionated using ZipTip C18 (Merck Millipore, Germany), according to the manufacturer’s protocol. The collected peptide samples were dried in a SpeedVac at room temperature and reconstituted in 15 µL of 0.1% trifluoroacetic acid (TFA). All peptide samples were then analyzed for the differential expression of peptide barcodes and peptide sequences using MALDI-TOF MS and ESI-LC-MS/MS.

### 4.6. MALDI-TOF MS and Peptide Barcode Interpretation

α-Cyano-4-hydroxycinnamic acid (CHCA) matrix (dissolved in 0.5% TFA and 50% acetonitrile) was mixed with peptide samples, and 1 µL was spotted onto a MALDI AnchorChip 384 target plate. To minimize bias, each sample was spotted in eight replicates. Four of the eight replicate spots were analyzed using the ClinProTools version 3.0 software to generate a pooled sample for each group. These pooled samples were spotted in 25 replicates to assess method reproducibility and were calibrated with a standard peptide mixture (mass range 1–20 kDa) from a ProteoMass Peptide & Protein MALDI-MS Calibration Kit (Sigma-Aldrich, St. Louis, MO, USA) as the external standard. The target plate was loaded into an Ultraflex III MALDI-TOF MS instrument (Bruker Daltonics, Bremen, Germany) immediately after matrix crystallization. Mass-spectral analysis was performed in linear positive mode with a mass range of 1000–10,000 Da and laser shots at 1500 shots/spot. External calibration was operated using standard peptide and protein mixtures, including human angiotensin II (*m*/*z* 1046), P14R (*m*/*z* 1533), human ACTH fragments 18–39 (*m*/*z* 2465), bovine insulin oxidized B chain (*m*/*z* 3465), and bovine insulin (*m*/*z* 5731) (ProteoMass™ Peptide and Protein MALDI-MS Calibration Kit; Sigma Aldrich, Burlington, MA, USA).

Mass spectra were acquired using an optimized method in flexControl version 3.0 (Bruker, Germany) and processed with flexAnalysis version 3.4 (Bruker, Germany). Candidate mass spectra were analyzed using three statistical algorithms incorporated into the ClinProTools software version 3.0: Quick Classifier (QC)/Genetic Algorithm-Based Different Average, Supervised Neural Network (SNN), and Genetic Algorithm (GA). Recognition capabilities and cross-validation values exceeding 90% were used to establish the reliability of candidate peak selection. Peak intensity values were then compared using three statistical tests also incorporated into ClinProTools: Anderson–Darling (AD), *t*-test/ANOVA (TTA), and Wilcoxon/Kruskal–Wallis (W/KW) [67]. Results with *p* < 0.05 were considered statistically significant.

### 4.7. Identification of Peptide Sequences by LC-MS/MS

Peptide samples were adjusted to 100 ng and injected into a nano-liquid chromatography–electrospray ionization–tandem mass spectrometry system (LC-ESI-MS/MS) for the analysis of gradient-eluted peptides. The LC-ESI-MS/MS system consisted of an UltiMate 3000 LC system (Dionex, Camberley, UK) coupled to a hybrid quadrupole Q-TOF impact IITM mass spectrometer (Bruker Daltonics, Bremen, Germany) equipped with a nano-captive spray ion source. The peptides were first enriched on a µ-precolumn (300 µm i.d. × 5 mm; C18 PepMap 100, 5 µm, 100 Å; Thermo Fisher Scientific, Loughborough, UK), followed by separation on a 75 µm i.d. × 15 cm column packed with Acclaim PepMap RSLC C18, 2 µm, 100 Å, nanoViper (Thermo Scientific, UK) at a flow rate of 300 nL/min for 30 min. Eluent A was 0.1% formic acid in water and eluent B was 80% acetonitrile in 0.1% formic acid. A gradient of 5–55% solvent B was used to elute the peptides at a constant flow rate of 0.30 μL/min for 30 min. Electrospray ionization was carried out at 1.6kV using the CaptiveSpray. Nitrogen was used as a drying gas (flow rate about 50 L/h). Collision-induced dissociation (CID) product ion mass spectra were obtained using nitrogen gas as the collision gas. Mass spectra (MS) and MS/MS spectra were obtained in positive ion mode at 2 Hz over the range (*m*/*z*) 150–2200. The collision energy was adjusted to 10 eV as a function of the *m*/*z* value. The LC-MS analysis of each sample was conducted in triplicate.

### 4.8. Data Processing and Statistical Analysis

The MaxQuant 1.6.1.12 software (http://maxquant.net, accessed on 10 June 2026) was used to evaluate all peptide masses. Quantitative peptide mass spectra were matched against the UniProt database for protein identification, with *Homo sapiens* selected as the taxonomy. Data were processed using a maximum of two missed cleavages, a mass tolerance of 20 ppm for the main search, an unspecific digestion mode, and variable modifications (methionine oxidation and N-terminal protein acetylation). The differential expression of peptides was analyzed using MetaboAnalyst (https://www.metaboanalyst.ca, accessed on 10 June 2026) and the Jvenn Venn diagram tool (http://jvenn.toulouse.inra.fr/app/index.html, accessed on 10 June 2026), accessed 10 June 2026. Peptides were then examined for interaction networks using STRING version 12.0 (https://string-db.org). Peptide intensities were presented as mean ± standard deviation (SD) for individual samples, and statistical significance (*p* < 0.05) was calculated using GraphPad Prism version 10.2.0 (GraphPad Software, San Diego, CA, USA) and IBM SPSS Statistics version 29.0.1.0 (IBM, Armonk, NY, USA).

For LC-MS/MS-derived peptides, differential expression between pre- and post-treatment timepoints was defined using a significance threshold of *p* < 0.05 from the Wilcoxon matched-pairs signed-rank test, without an additional fixed fold-change cutoff. Because this analysis was exploratory and hypothesis-generating across a panel of differentially detected peptides, *p*-values were not corrected for multiple comparisons; the findings should therefore be interpreted as hypothesis-generating pending confirmation in independent cohorts.

## 5. Conclusions

**Figure 12:**
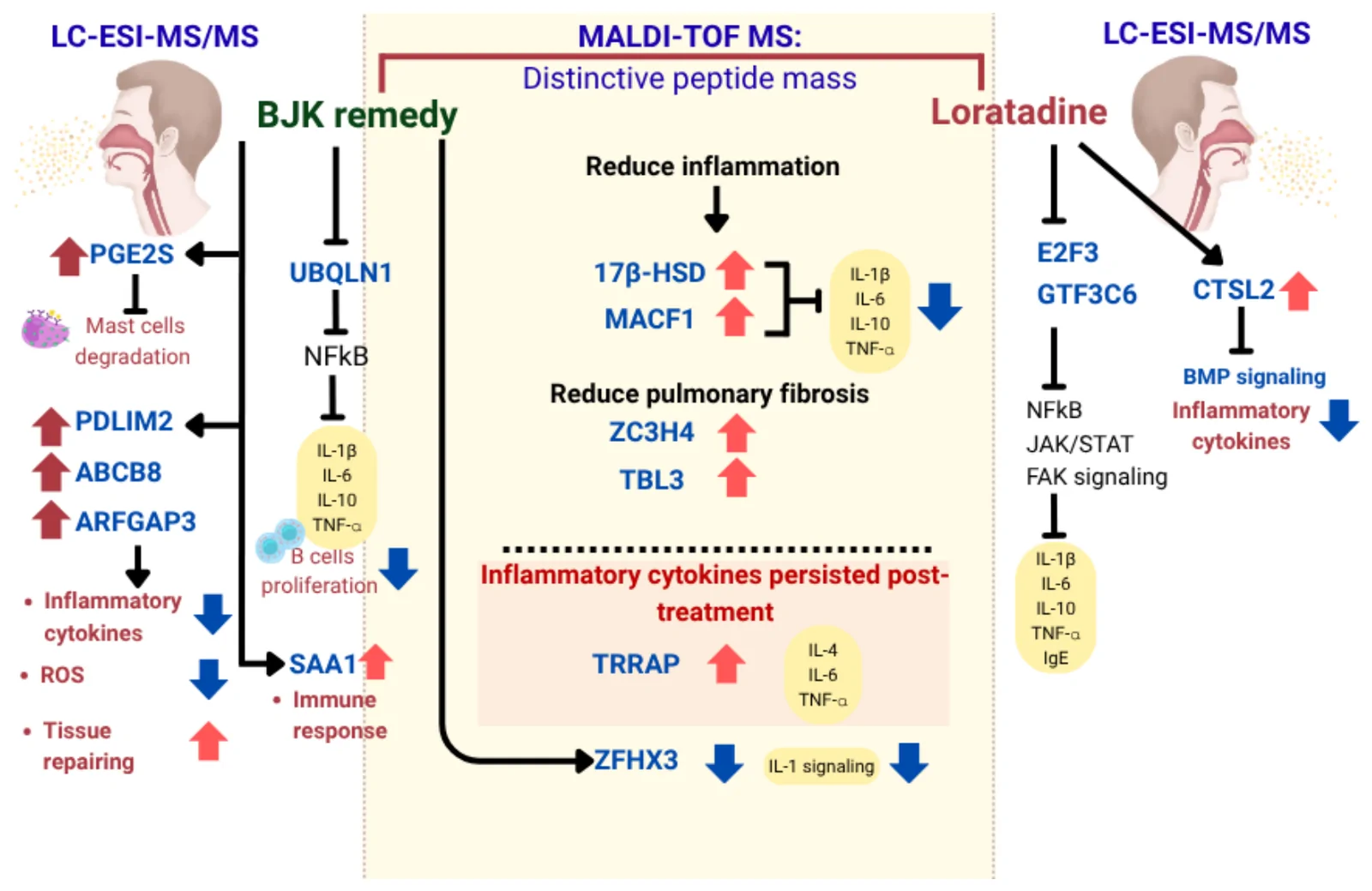
Schematic summary of characteristic peptide profiles after administration of BJK remedy and loratadine.

In summary, both BJK remedy and loratadine may share a common mechanism of alleviating AR symptoms through the upregulation of 17β-HSD and MACF1. Both treatments may also reduce pulmonary fibrosis and support tissue repair by increasing ZC3H4 and TBL3. Meanwhile, ZFHX3 was decreased at post-treatment with BJK remedy, supporting the potential anti-inflammatory effect through the modulation of its expression. Conversely, TRRAP levels remained elevated after treatment, similar to the pre-treatment values. Because there is no permanent cure for AR, symptom recurrence is likely upon re-exposure to allergens. The LC-MS/MS analysis of distinct peptide patterns revealed two principal pathways: the suppression of allergic inflammation and the enhancement of immune balance and tissue remodeling. BJK remedy reduced inflammation by downregulating UBQLN1, upregulating PTGES2 and PDLIM2, promoting antioxidant activity, and immune modulation through ABCB8 and ARFGAP3. Loratadine, in contrast, suppressed E2F3 and GTF3C6 and increased CTDSPL2 expression, indicating a distinct yet complementary anti-inflammatory effect (Figure 12). These findings enhance our understanding of AR pathophysiology and support further research aimed at validating these proteins in larger cohorts to develop targeted therapies or biomarkers. To our knowledge, this is the first report describing the proteomic mechanisms of BJK and loratadine in AR; both compounds suppressed inflammatory cytokine pathways, but BJK additionally promoted tissue repair and antioxidant activity. These results also support the use of BJK in the treatment of conditions ascribed to water- and earth-element diseases in Thai traditional medicine.

## Figures and Tables

**Table 1:** Comparative baseline characteristics of allergic rhinitis patients in the two treatment groups.

Characteristic	BJK Treatment Group	Loratadine Treatment Group	*p*-Value
Age (years)	30.17 ± 7.48	33.86 ± 11.95	0.426
Gender: male (n)	8.70%	13.04%	0.726
Gender: female (n)	41.30%	36.96%	
Weight (kg)	59.52 ± 7.48	59.63 ± 11.03	0.968
Height (cm)	161.08 ± 7.07	161.69 ± 6.31	0.778
BMI	22.88 ± 3.83	22.72 ± 3.52	0.894
Total nasal symptom score (TNSS)	6.59 ± 1.86	7.17 ± 1.46	0.291

Comparisons were performed using the Mann–Whitney U test and unpaired test (independent *t*-test) as appropriate. A *p*-value < 0.05 was considered statistically significant. Data are presented as mean ± SD. BMI, body mass index; TNSS, total nasal symptom score; *n* = 23.

**Table 2:** Detailed identification of distinctive peptide masses matched against the UniProt database.

Mass (Da)	Protein Name	Protein ID	Sequence	Score	Function
1225.66	17beta-hydroxysteroid dehydrogenase type 7 form 2 (17β-HSD)	Q5MGS6	ITDFHICKCF	19.777	Selectively catalyzes this conversion and is expressed in steroidogenic and peripheral tissues, consistent with a local intracrine role [13].
1364.31	Microtubule actin crosslinking factor 1 (MACF1)	A0A0A6YYJ5	VPQRSIYDTMR	11.593	Regulates Wnt/catenin signaling in osteogenesis [14].
1465.67	Zinc finger homeobox protein 3 (ZFHX3)	Q15911	RPQLPVSDRHVY	1.499	Associated with atrial fibrillation [15].
1486.82	Transformation/transcription domain-associated protein (TRRAP)	Q9Y4A5	ALWQIPPVGICYK	7.544	Binds directly to the E2F1 transactivation domain and is required, together with c-Myc, for oncogenic transformation; this direct interaction was demonstrated by the co-immunoprecipitation of endogenous complexes [16].
1544.21	Zinc finger CCCH domain-containing protein 4 (ZC3H4)	Q9UPT8	DEKGMDEEEDGEY	4.705	Regulates infiltrating monocytes and attenuates pulmonary fibrosis through IL-10 [17].
2115.84	Transducin beta-like protein 3 (TBL3)	A0JLS5	VILWKDVTEAEQAEEQAR	16.123	Regulates transcription of P53 gene. Regulates ribosome synthesis [18].

**Table 5:** Fold changes and detection frequencies of peptides between pre- and post-treatment with BJK remedy.

Protein Name	Log 2 Intensity						Fold Change	*p*-Value	McNemar Test *p*-Value	Fold Change	*p*-Value	McNemar Test *p*-Value
	AR	Detection Frequency	ARB3	Detection Frequency	ARB6	Detection Frequency	(ARB3:AR)			(ARB6:AR)		
**UBQLN1**	6.98 ± 6.35	13/23 (56.52%)	4.97 ± 7.13	8/23 (34.78%)	6.98 ± 6.35	4/23 (17.39%)	−2.01	0.326	0.146	−4.562	0.016 *	0.012 ^#^
**CLIC2**	4.72 ± 6.08	9/23 (39.13%)	4.19 ± 5.93	8/23 (34.78%)	6.86 ± 6.85	12/23 (52.17%)	−0.529	0.865	0.791	2.134	0.196	0.549
**UCN3**	5.34 ± 6.31	10/23 (43.47%)	5.43 ± 6.39	10/23 (43.47%)	7.91 ± 6.08	15/23 (65.21%)	0.093	0.861	1.000	2.564	0.414	0.332
**PTGES2**	1.23 ± 4.08	2/23 (8.69%)	4.42 ± 6.27	8/23 (34.78%)	9.07 ± 7.02	15/23 (65.21%)	3.189	0.110	0.180	7.841	0.004 **	0.002 ^##^
**SNF8**	4.58 ± 5.93	9/23 (39.13%)	6.09 ± 6.64	11/23 (47.82%)	6.78 ± 6.69	12/23 (52.17%)	1.514	0.332	1.000	2.203	0.753	0.549
**IRF-6**	5.26 ± 6.15	10/23 (43.47%)	5.46 ± 6.44	10/23 (43.47%)	8.28 ± 5.64	16/23 (69.56%)	0.205	0.875	1.000	3.024	0.102	0.249
**SAA1**	5.24 ± 6.14	10/23 (43.47%)	4.52 ± 6.36	8/23 (34.78%)	8.11 ± 6.07	15/23 (65.21%)	−0.713	0.910	0.581	2.870	0.044 *	0.180
**METTL26**	4.36 ± 6.13	8/23 (34.78%)	2.67 ± 5.20	5/23 (21.73%)	8.21 ± 7.60	13/23 (56.52%)	−1.687	0.203	0.289	3.852	0.047 *	0.180
**PDLIM2**	3.79 ± 6.75	6/23 (26.08%)	1.23 ± 4.08	10/23 (43.47%)	12.49 ± 8.19	17/23 (73.91%)	2.084	0.382	0.549	8.697	0.002 **	0.003 ^##^
**ADCK5**	4.99 ± 6.41	9/23 (39.13%)	5.71 ± 6.41	10/23 (43.47%)	9.44 ± 5.21	18/23 (78.26%)	0.724	0.683	1.000	4.444	0.052	0.022 ^#^
**ABCB8**	3.47 ± 5.42	7/23 (30.43%)	6.11 ± 6.59	11/23 (47.82%)	8.50 ± 6.64	16/23 (69.56%)	2.636	0.064	0.549	5.028	0.003 **	0.008 ^##^
**ARFGAP3**	4.79 ± 6.87	8/23 (34.78%)	6.87 ± 7.66	11/23 (47.82%)	11.46 ± 6.62	18/23 (78.26%)	2.079	0.326	0.791	6.666	0.005 **	0.006 ^##^
**MPP4**	6.08 ± 6.60	11/23 (47.82%)	5.02 ± 6.46	9/23 (39.13%)	6.51 ± 6.43	12/23 (52.17%)	−1.064	0.570	0.549	0.425	0.948	1.000
**MAD2L1BP**	5.74 ± 6.82	10/23 (43.47%)	5.89 ± 6.94	10/23 (43.47%)	8.16 ± 6.78	14/23 (60.86%)	0.144	0.820	1.000	2.421	0.196	0.289

Data are demonstrated as mean ± SD. Peptide detection frequency is expressed as detected samples/total sample (%). Peptide intensity was analyzed using the Wilcoxon matched-pairs signed-rank test *p*-value < 0.05 (*); *p*-value < 0.01 (**), detection frequency was analyzed using McNemar’s test *p*-value < 0.05 (^#^); *p*-value < 0.01 (^##^); *n* = 23.

**Table 6:** Fold changes and detection frequencies of peptides between pre- and post-treatment with loratadine.

Protein Name	Log 2 Intensity						Fold Change	*p*-Value	McNemar Test *p*-Value	Fold Change	*p*-Value	McNemar Test *p*-Value
	AR	Detection Frequency	ARL3	Detection Frequency	ARL6	Detection Frequency	(ARL3:AR)			(ARL6:AR)		
**BKRB2**	7.87 ± 7.28	13/23 (56.52%)	4.83 ± 6.86	8/23 (34.78%)	6.54 ± 7.11	11/23 (47.82%)	−3.041	0.112	0.180	−1.329	0.445	0.791
**E2F3**	8.24 ± 7.60	13/23 (56.52%)	4.20 ± 5.89	8/23 (34.78%)	6.55 ± 7.18	11/23 (47.82%)	−4.041	0.034 *	0.227	−1.689	0.55	0.804
**GTF3C6**	9.74 ± 7.56	15/23 (65.21%)	4.14 ± 6.49	7/23 (30.43%)	5.98 ± 7.07	10/23 (43.47%)	−5.595	0.017 *	0.039 ^#^	−3.757	0.616	0.302
**SAA1**	4.72 ± 6.04	9/23 (39.13%)	7.06 ± 6.42	13/23 (56.52%)	3.58 ± 5.54	7/23 (30.43%)	2.349	0.309	0.388	−1.138	0.345	0.754
**FAM181B**	5.91 ± 6.54	11/23 (47.82%)	8.88 ± 6.17	16/23 (69.56%)	4.47 ± 6.08	9/23 (39.13%)	2.688	0.171	0.227	−1.459	0.334	0.754
**MEF2A**	4.37 ± 6.13	8/23 (34.78%)	4.85 ± 6.20	9/23 (39.13%)	7.97 ± 6.01	15/23 (65.21%)	0.482	0.937	1.000	3.598	0.091	0.118
**MECP2**	5.47 ± 5.93	11/23 (47.82%)	5.08 ± 6.72	9/23 (39.13%)	7.40 ± 5.56	15/23 (65.21%)	−0.392	0.943	0.791	1.921	0.243	0.388
**PSG3**	6.32 ± 6.83	11/23 (47.82%)	5.70 ± 6.12	11/23 (47.82%)	6.05 ± 6.04	12/23 (52.17%)	−0.624	0.435	1.000	−0.267	0.619	1.000
**CTDSPL2**	4.70 ± 6.04	9/23 (39.13%)	9.51 ± 6.11	17/23 (73.91%)	10.87 ± 4.61	20/23 (86.95%)	0.272	0.03 *	0.039 ^#^	2.035	0.005 **	0.007 ^##^
**GRHL3**	6.30 ± 6.85	11/23 (47.82%)	4.31 ± 6.80	7/23 (30.43%)	6.56 ± 6.51	12/23 (52.17%)	−1.997	0.301	0.424	0.254	0.811	1.000

Data are demonstrated as mean ± SD. Peptide detection frequency is expressed as detected samples/total sample (%). Peptide intensity was analyzed using the Wilcoxon matched-pairs signed-rank test *p*-value < 0.05 (*); *p*-value < 0.01 (**), detection frequency was analyzed using McNemar’s test *p*-value < 0.05 (^#^); *p*-value < 0.01 (^##^); *n* = 23.

## Data Availability

The dataset is not publicly available due to privacy considerations, but data from this study are available from the corresponding author upon reasonable request.

## Supplementary Materials

The following supporting information can be downloaded at: https://www.mdpi.com/article/10.3390/ijms27167389/s1.

## Abbreviations

The following abbreviations are used in this manuscript:

ARF1	ADP-ribosylation factor 1
AR	Allergic rhinitis
ACTN4	Actinin alpha 4
APC	Adenomatous polyposis coli
BJK	Benjakul
CDKN1A	Cyclin-dependent kinase inhibitor 1A
CHCA	α-Cyano-4-hydroxycinnamic acid
COPB1	COPI coat complex subunit beta 1
CCDC51	Coiled-coil domain containing 51
E2F1	E2F transcription factor 1
E2F4	E2F transcription factor 4
ESI-LC-MS/MS	Nano-liquid chromatography–electrospray ionization–tandem mass spectrometry
ESR2	Estrogen receptor
GSK3B	Glycogen synthase kinase-3 beta
HES1	Hairy and enhancer of split-1
IFN-γ	Interferon gamma
IL-1β	Interleukin 1 beta
IL-4	Interleukin 4
IL-6	Interleukin 6
IL-10	Interleukin 10
MALDI-TOF MS	Matrix-assisted laser desorption/ionization time-of-flight mass spectrometry
METTL25	Methyltransferase-like 25
MYO6	Myosin 6
NOTCH1	Neurogenic locus notch homolog protein 1
NTMT1	N-terminal methyltransferase 1
PDLIM1	PDLIM1-interacting kinase 1-like
POU1F1	E3 SUMO-protein ligase PIAS3 and pituitary-specific positive transcription factor 1
RBBP6	E3 ubiquitin-protein ligase RBBP6
RUNX3	Runt-related transcription factor 3
RPS19BP1	Active regulator of SIRT1
SLC25A28	Mitoferrin 2
STK35	Serine/threonine kinase 35
TCF7	General transcription factor 3C complex, transcription factor 7
TP53	Tumor protein p53
TNF-α	Tumor necrosis factor alpha
WDR36	WD repeat-containing protein 36.
