# Supplementary material for: Serum Peptidomic Analysis in a Comparative Study of the Efficacy of Benjakul Remedy Versus Loratadine in Allergic Rhinitis Patients
Source: Int J Mol Sci. 2026 Aug 18;27(16):7389. doi: 10.3390/ijms27167389 (PMC13513288; doi:10.3390/ijms27167389)

Supplementary Materials

Content:

Figure S1. HPLC profiling of the BJK extract demonstrated the several bioactive constituents: piperine was the major constituent in extract (with concentration of 97.46 ± 6.06 mg/g extract), follow by 6-shogaol, 6-gingerol, myristicin, plumbagin, and methyl piperate , respectively (with concentration of 12.00 ± 0.46, 11.27 ± 0.76, 6.83 ± 0.25, 5.55 ± 0.40, and 3.07 ± 0.15 mg/g extract).

BJK extract

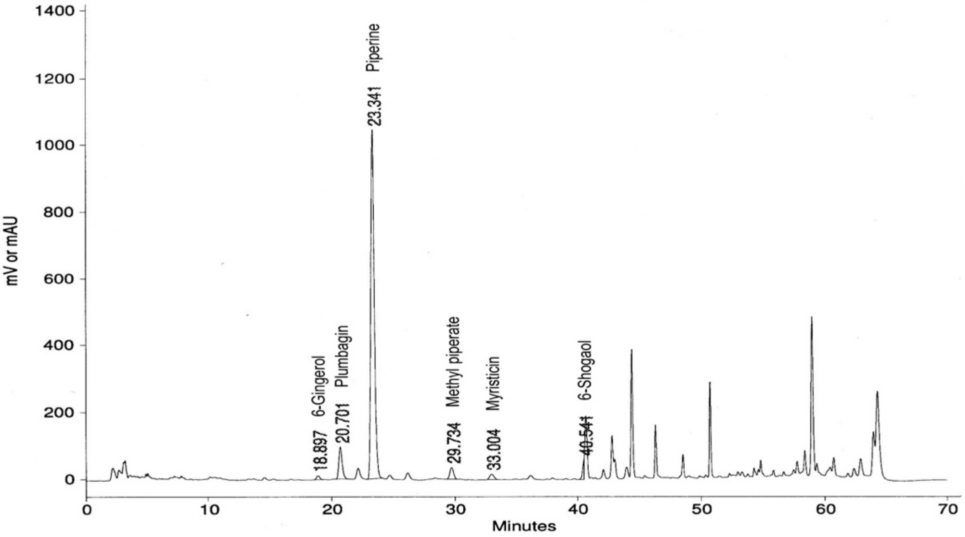

Standard

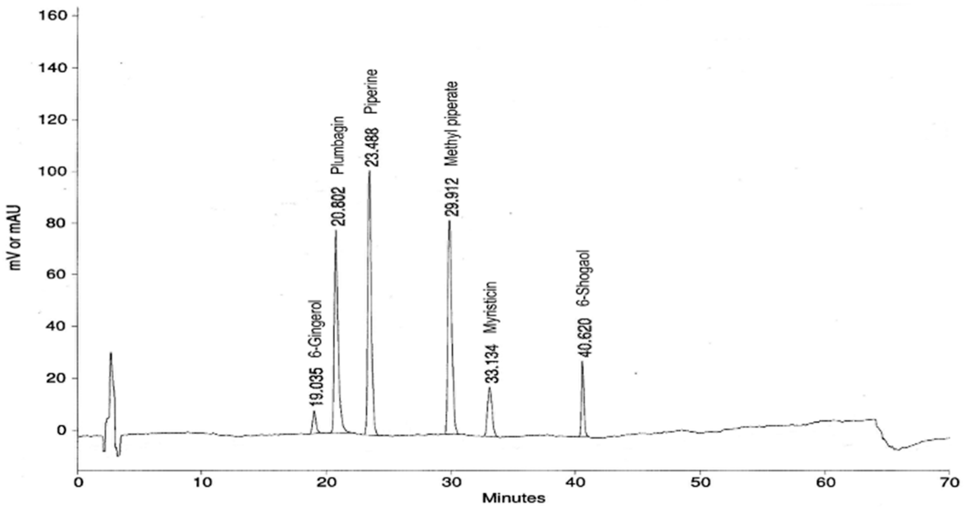

Supplement: Supplementary file 1 [file ijms-27-07389-s001.zip › ijms-4458300-supplementary.pdf]
